# Simultaneous CRISPR/Cas9 Editing of Three PPO Genes Reduces Fruit Flesh Browning in *Solanum melongena* L.

**DOI:** 10.3389/fpls.2020.607161

**Published:** 2020-12-03

**Authors:** Alex Maioli, Silvia Gianoglio, Andrea Moglia, Alberto Acquadro, Danila Valentino, Anna Maria Milani, Jaime Prohens, Diego Orzaez, Antonio Granell, Sergio Lanteri, Cinzia Comino

**Affiliations:** ^1^DISAFA, Plant Genetics and Breeding, University of Torino, Grugliasco, Italy; ^2^Crop Biotechnology Department, Instituto de Biología Molecular y Celular de Plantas (IBMCP), CSIC-UPV, Valencia, Spain; ^3^Instituto de Conservación y Mejora de la Agrodiversidad Valenciana, Universitat Politècnica de València, Valencia, Spain

**Keywords:** gene editing, CRISPR/Cas 9, eggplant, polyphenol oxydase, knock-out

## Abstract

Polyphenol oxidases (PPOs) catalyze the oxidization of polyphenols, which in turn causes the browning of the eggplant berry flesh after cutting. This has a negative impact on fruit quality for both industrial transformation and fresh consumption. Ten *PPO* genes (named *SmelPPO1*-*10*) were identified in eggplant thanks to the recent availability of a high-quality genome sequence. A CRISPR/Cas9-based mutagenesis approach was applied to knock-out three target *PPO* genes (*SmelPPO4, SmelPPO5*, and *SmelPPO6)*, which showed high transcript levels in the fruit after cutting. An optimized transformation protocol for eggplant cotyledons was used to obtain plants in which Cas9 is directed to a conserved region shared by the three *PPO* genes. The successful editing of the *SmelPPO4, SmelPPO5*, and *SmelPPO6* loci of *in vitro* regenerated plantlets was confirmed by Illumina deep sequencing of amplicons of the target sites. Besides, deep sequencing of amplicons of the potential off-target loci identified *in silico* proved the absence of detectable non-specific mutations. The induced mutations were stably inherited in the T_1_ and T_2_ progeny and were associated with a reduced PPO activity and browning of the berry flesh after cutting. Our results provide the first example of the use of the CRISPR/Cas9 system in eggplant for biotechnological applications and open the way to the development of eggplant genotypes with low flesh browning which maintain a high polyphenol content in the berries.

## Introduction

The polyphenol oxidases (PPOs) are a group of enzymes catalyzing the oxidation of phenolic compounds into highly reactive quinones (Prohens et al., 2007; Mishra et al., 2013; Plazas et al., 2013; García-Fortea et al., 2020). The physiological role of PPOs in plants has not been fully clarified yet, but a defense role against pathogens and pests has been postulated because of their increased localized activity in response to cutting and wounding. The relationship between PPO expression or activation and pathogen infections was proved in tomato by either silencing (Thipyapong et al., 2004) or over-expressing PPO genes (Li and Steffens, 2002). PPOs oxidize polyphenols to toxic quinones which bind to amino acids in the insect gut, exerting an anti-feeding role. Previous studies have associated PPO activity with resistance to various types of insects (Mahanil et al., 2008).

In recent years, PPOs have been largely investigated for their involvement in the browning process, a color reaction caused by the oxidation of phenolic compounds during postharvest processing and storage. Enzymatic browning is a two-step reaction, consisting of the oxidation of a monophenol to a o-diphenol (cresolase/monophenolase activity), which is further oxidized to yield a o-quinone (catecholase/diphenolase activity). O-quinones can then undergo condensation or polymerization reactions, producing the dark pigments melanins. Fruit cutting causes cellular disruption and damages membrane integrity, allowing the PPOs sequestered in the plastid to come into contact with the hydroxycinnamic acid derivatives, which are their substrates. Extensive browning of cut fruit and vegetable surface compromises food quality and usually impairs the properties of the product, representing a major economic problem both for the food industry (e.g., the industrial manipulation and preservation of these products) and for consumers (in the case of fresh and ready-to-eat fresh cut fruit and vegetables). Since PPO activity is influenced by factors such as pH, temperature and oxygen, the browning process is limited in the food industry through the use of chemical and/or physical agents, with a negative impact on nutritional and organoleptic properties. Browning negatively affects the commercial value of many key agricultural productions, including potato, lettuce, cereals, banana, cucumber, grape and eggplant (Taranto et al., 2017).

Eggplant (*Solanum melongena* L.) berries are characterized by a remarkable content in phenolic compounds, represented mainly by chlorogenic acid (5-O-caffeoylquinic acid). Chlorogenic acid plays important therapeutic roles due to its antioxidant, antibacterial, hepatoprotective, cardioprotective, anti-inflammatory and anti-microbial properties (Naveed et al., 2018). In eggplant, a correlation between the concentration of phenolics (mainly chlorogenic acid) and browning has been detected in the fruit flesh, although additional morphological and physiological factors may be involved in browning phenomena (Kaushik et al., 2017). Furthermore, in commercial varieties, the selection for berries with a reduced degree of browning in the flesh has resulted in the indirect selection of accessions with lower concentrations of phenolics (Prohens et al., 2007).

Shetty et al. (2011) identified six genes encoding PPOs in eggplant and, on the basis of both protein sequence similarity and organ-specific patterns of expression, they proposed the distinction of eggplant PPOs in two clades: A and B, with clade A encompassing genes expressed mostly in roots, while clade B genes are involved in defense mechanisms. This categorization was further extended to the rest of Solanaceae PPOs (Taranto et al., 2017).

The development of new technologies to disable genes coding for PPOs represents the most promising strategy to avoid undesired browning in plant-derived products, as it would allow to positively select genotypes enriched in beneficial phenolic compounds, while reducing the need for physical and chemical treatments in the food industry. The positive impact on the storability of these foods, in addition, would help reduce waste.

Several examples are available on the adoption of RNA silencing strategies to down-regulate PPO genes in order to reduce the enzymatic browning in potato tubers (Bachem et al., 1994; Coetzer et al., 2001; Rommens et al., 2006; Llorente et al., 2011; Chi et al., 2014). By using artificial micro-RNAs (amiRNAs) all *StuPPO* genes have been silenced individually or in combination, identifying *StuPPO*_2_ as the main contributor to PPO activity (Chi et al., 2014). A few notable examples exist of commercially available genetically modified plants in which PPOs have been silenced, such as the Arctic Apple® and the Innate® potato. The emergent CRISPR/Cas9 technology has proved extremely efficient in gene editing and is expected to play a key role in crop breeding. This technology makes it possible to induce point mutations in one or multiple target sequences simultaneously, as well as to introduce new genetic variants through homology directed recombination (HDR), or to modulate transcription and chromatin structure at selected target loci (Doudna and Charpentier, 2014). While this technique has been successfully applied to some Solanaceae species, such as tomato and potato, including the knock out of the *StuPPO2* gene in the potato tetraploid cultivar Desiree (González et al., 2020), no examples of genome editing in eggplant have been reported in literature so far (Van Eck, 2018).

In this study, thanks to the recent availability of a high quality, annotated and anchored eggplant genome sequence (https://solgenomics.net/organism/Solanum_melongena/genome; Barchi et al., 2019), we report the homology-based characterization, functional domain identification and phylogenetic analysis, of 10 PPO (*SmelPPO1-PPO10*) genes in eggplant. On the basis of their expression in the fruit after cutting, *SmelPPO4, SmelPPO5*, and *SmelPPO6* were selected for the generation of knock-out mutants using the CRISPR/Cas9 technology. Regenerated T_0_, T_1_, and T_2_ lines were screened for induced mutations in the target genes as well as in potential off-target loci. In addition, PPO activity and the degree of browning in the flesh of eggplant berries were analyzed in our knock-out T_1_ and T_2_ edited lines.

## Materials and Methods

### Mining of PPO in the Eggplant Genome and Phylogenetic Analysis

The six eggplant PPO aminoacidic sequences previously reported (Shetty et al., 2011) were used for a BlastP search of the eggplant proteome (https://solgenomics.net/organism/Solanum_melongena/genome) with an E-value threshold of 1 e^−5^. The polypeptide sequences of eggplant PPOs, together with those of six tomato and nine potato PPOs (Data Sheet 1), were used for a multiple alignment (Clustal Omega; https://www.ebi.ac.uk/Tools/msa/clustalo/). A phylogenetic analysis was performed with the MEGA X software. An unrooted phylogenetic tree was generated, applying the Neighbor-Joining (NJ) algorithm. The statistical significance of individual nodes was assessed by bootstrap analysis with 1,000 replicates, and the evolutionary distances were calculated using the p-distance method with default parameters.

### qPCR Analysis

To identify *PPO* genes involved in the browning phenotype their corresponding mRNA levels were analyzed in the flesh of fruits of the “Black Beauty” variety harvested at the commercial ripening stage (Mennella et al., 2012) after cutting them transversally with a sharp knife. One gram of frozen fruit flesh was ground in liquid nitrogen to a fine powder and RNA was extracted using the “Spectrum plant total RNA kit” (Sigma-Aldrich, St. Louis, USA). RNA was extracted in three biological replicates from commercial grade ripe fruit 1 cm-wide slices exposed to air for 0 min (t_0_) and 30 min (t_30_).

cDNA was synthesized from 1 μg of RNA using a High Capacity RNA-to-cDNA kit (Applied Biosystems, Foster City, USA) as directed by the manufacturer. Using the Primer 3 software (http://bioinfo.ut.ee/primer3), primers targeting the ten identified eggplant *PPO* genes were designed (Supplementary Table 1). PCR reactions were carried out in three biological replicates using the StepOnePlus Real-Time PCR System (Applied Biosystems). The following PCR program was used: 95°C/10 min, followed by 40 cycles of 95°C/15 s and 60°C/1 min. Data were quantified using the 2^−ΔΔCt^ method based on Ct values of actin and elongation factor as housekeeping genes. Values are expressed as relative mRNA abundance at 30 min after cutting compared to time 0 (just after cutting).

### Target Identification, DNA Construct Cloning, and Off-Target Search

Sequences of the wound-induced *SmelPPO4, SmelPPO5*, and *SmelPPO6* genes were aligned to find conserved regions, and BlastX and Prosite were used to annotate functional domains. A gRNA (ATGAATGGAAAGCAATCGGA) was designed to target a conserved region of these three genes and assembled into a CRISPR/Cas9 construct carrying the h*Cas9* and the *nptII* gene for kanamycin resistance, using the GoldenBraid (GB) assembly system and following GB software-directed procedures (https://gbcloning.upv.es/). An additional guanine was added at the 5'end in order to improve expression under the U6-26 RNA PolIII promoter (Cong and Zhang, 2015). The h*Cas9* expression is driven by the CaMV 35S promoter, while the gRNA is placed under the control of the *At*U6-26 RNA PolIII promoter. Putative off-target sites were identified with the CasOT software (http://casot.cbi.pku.edu.cn/), using the eggplant genome as reference. Four off-targets (OT1-OT4) were selected based on the number and position of mismatches (Supplementary Table 2); the corresponding loci (a 1 kb region around the putative off-target site) were inspected, to determine whether they corresponded to functional genes, and considered for sequencing analyses.

### Genetic Transformation of Plants

The final pCambia vector *Tnos:nptII:Pnos-U6-26:gRNA:scaffold-P35S:*h*Cas9:Tnos* was transformed into LBA4404 *Agrobacterium tumefaciens* strain. A pre-culture was set up in a modified MGL liquid medium (tryptone 5 g l^−1^, yeast extract 2.5 g l^−1^, NaCl 0.1 g l^−1^, mannitol 5 g l^−1^, glutamic acid 1.15 g l^−1^, KH_2_PO_4_ 0.25 g l^−1^, MgSO_4_.7H_2_O 100 g l^−1^, biotin 1 mg l^−1^, pH 7) supplemented with 50 mg l^−1^ rifampicin and 50 mg l^−1^ kanamycin, and incubated overnight at 28°C. From this, a second culture was set up in TY liquid medium (tryptone 5 g l^−1^, yeast extract 3 g l^−1^, MgSO_4_.7H_2_O 0.5 g l^−1^, pH 5.8) supplemented with 200 μM acetosyringone and incubated overnight in the dark at 28°C. Before transformation, the optical density of the culture at 600 nm (OD_600_) was measured and the bacterial culture was diluted to a final OD_600_ of 0.10–0.15 in TY medium supplemented with 200 μM acetosyringone. Explants of about 5 mm in length were cut from the cotyledons of *in vitro* germinated ‘Black Beauty’ seeds, dipped in the bacterial culture for a minimum of 10 min, blotted dry on filter paper and transferred for 48 h on a co-culture medium (MS basal salt mixture 4.5 g l^−1^, MES 0.5 g l^−1^, sucrose 30 g l^−1^, phytoagar 10 g l^−1^, Gamborg vitamin mixture 1 ml l^−1^, trans-zeatin 2 mg l^−1^, IAA 0.1 mg l^−1^, acetosyringone 200 μM, pH 5.8), in the dark. For organogenesis and shoot induction, a common basal induction medium was used, as previously described (Muktadir et al., 2016) (MS basal salt mixture 4.5 g l^−1^, MES 0.5 g l^−1^, sucrose 30 g l^−1^, phytoagar 10 g l^−1^, Gamborg vitamin mixture 1 ml l^−1^, trans-zeatin 2 mg l^−1^, IAA 0.1 mg l^−1^, kanamycin 30 mg l^−1^, carbenicillin 400 mg l^−1^, pH 5.8), with three different conditions: without further additives, with supplementation of ascorbic acid 5 mg l^−1^ and citric acid 5 mg l^−1^, and with supplementation of polyvinylpyrrolidone (PVP40) 200 mg l^−1^. Furthermore, for each medium composition, two conditions were tested during the first 3 days of induction: no incubation, or 3 days of incubation in the dark, after which explants were grown in the same conditions as the untreated group (16:8 light:dark cycle, 24°C). Elongation and rooting were performed on the same media for all conditions and explants were moved to a fresh medium every 2–3 weeks. Both media were previously described (Muktadir et al., 2016) and were not supplemented with antioxidants, as no oxidative damage was observed from this stage onwards. The elongation medium was supplemented with kanamycin 30 mg l^−1^ and carbenicillin 400 mg l^−1^, but did not contain any hormone. Kanamycin was removed from the rooting medium to avoid inhibitory effects on root development, and 0.2 mg l^−1^ indolebutyric acid were added. Fully developed plantlets were then moved to soil and gradually acclimated to *ex vitro* conditions.

### Target and Off-Target Sequencing

Genomic DNA was extracted using a CTAB protocol (Doyle and Doyle, 1987) from leaves sampled when plantlets were transferred from *in vitro* growth conditions to soil. The presence of the transgene was assessed by amplifying the h*Cas9* (Supplementary Table 3) gene by using qPCR (in three technical replicates) according to the protocol described in the previous paragraph. DNA was also extracted from T_1_ and T_2_ progeny plants (Data Sheet 3).

Mutation frequencies at the target and off-target sites were evaluated according to an adapted version of the 16S Metagenomic Sequencing Library preparation protocol provided by Illumina (16S Sample Preparation Guide). Amplifications were carried out using the KAPA HiFi HotStart ReadyMix PCR Kit (Kapa Biosystems, Boston, MA). Dual indexing was done using the Nextera XT system (Illumina, San Diego, CA) using 16 i5 indexes (S502-S522) and 24 i7 indexes (N701-N729), enabling the multiplexing of 333 individual libraries. Due to their high sequence identity, a differential amplification of *SmelPPO4* and *SmelPPO6* was obtained with a first specific PCR, using primers designed on flanking non conserved regions, while a second amplification was performed with non-specific primers carrying Illumina adapter sequences (Supplementary Table 3). Amplifications of *SmelPPO5*, OT1, OT2, OT3, OT4 were done directly using primers modified with Illumina adapter sequences (Supplementary Table 3). Products were diluted 1:50 and used as templates to add dual Nextera XT indexes (Data Sheet 2). Finally, indexed amplicons were purified using AmpureBeads (0,7X) and quantified using Qubit 2.0 (Life Technologies, Carlsbad, CA, USA), based on the Qubit dsDNA HS Assay (Life Technologies, Carlsbad, CA, USA). All samples were diluted to 4 nM and pooled in a single tube. Sequencing was performed with an Illumina MiSeq sequencer (Illumina Inc., San Diego, CA) and 150 bp paired-end reads were generated. From reads generated by WGS sequencing, adapters were removed and reads that were <50 nucleotides long were discarded using Trimmomatic v0.39 (Bolger et al., 2014). Processed reads were analyzed for CRISPR/Cas9 editing events with CRISPResso2 [http://crispresso2.pinellolab.org (Clement et al., 2019)] (Supplementary Table 4). Sequences can be can be downloaded at https://www.crispr-plants.unito.it/eggplant.

### PPO Activity Assay

Fruits of the wild type and edited lines (T_1_ and T_2_) were collected in eight biological replicates at the commercial ripening stage (Mennella et al., 2012). Flesh slices about 1 cm thick, cut at the midpoint between the blossom and stem ends, were exposed to air for 30 min (t_30_) before pictures were taken. After the exposition, all fresh tissues were immediately frozen in liquid nitrogen and stored at −80°C for PPO activity measurement of the eight biological replicates. PPO activity analysis was performed according to previously described protocols (Bellés et al., 2006; Plazas et al., 2013) with minor modifications: 1 g of fresh frozen peel tissue was taken and ground in a mortar with liquid nitrogen and 50 mg of polyvinylpolypyrrolidone before being resuspended in 4 ml 0.1 M sodium phosphate buffer pH 6. Samples were sonicated in a water bath for 10′ at 20°C, centrifuged at 12,000 rpm for 15′ at 4°C and the supernatant was collected. Protein concentration was evaluated using Bradford's dye (Sigma Aldrich) binding assay using bovine serum albumin (Sigma Aldrich) as a standard (Bradford, 1976). PPO activity was measured colorimetrically at room temperature using a spectrophotometer (Beckman Coulter, Brea, CA, USA) to follow the emerging enzymatic reaction. For sample analysis, 145 μl sodium phosphate buffer (0.1 M, pH 6, RT), 15 μl chlorogenic acid (Sigma Aldrich, 35.5 mg ml^−1^), and 40 μl of protein extract were mixed and absorbance (415 nm) measured every 10 s for 25 min. A negative control without protein extract was even analyzed. One unit of enzyme activity was defined as the increase in 0.1 absorbance unit per minute per milligram of fresh weight (Kaushik et al., 2017).

## Results and Discussion

### *SmelPPO* Identification and Phylogenetic Analysis

**Figure 1:**
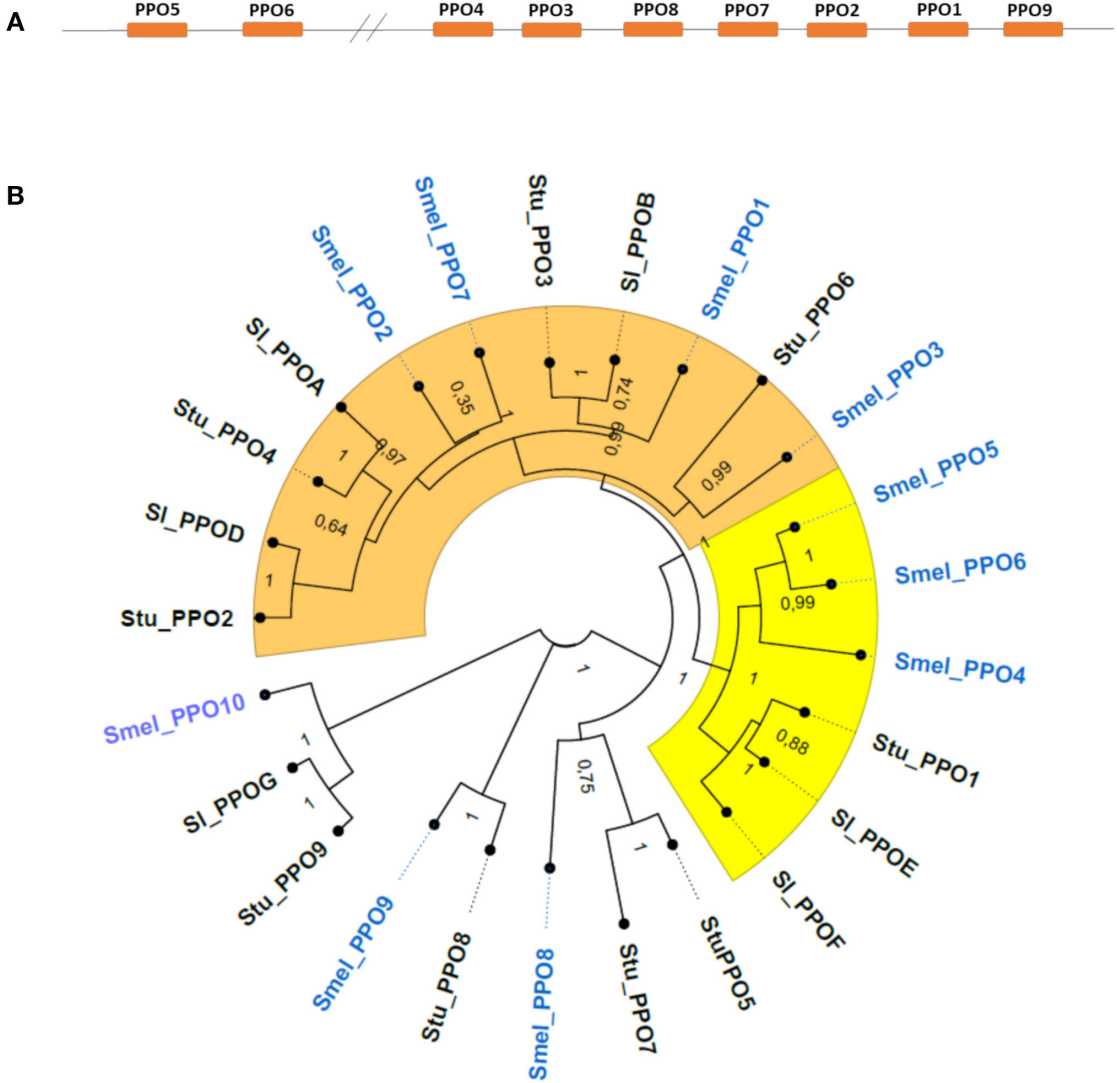
**(A)** Relative position and organization of *PPO1-9* genes on chromosome 8 of *Solanum melongena*. All eggplant *PPO*s except *SmelPPO10* are located on chromosome 8. **(B)** Phylogenetic analysis of PPO proteins. The neighbor-joining trees was constructed by aligning the PPO protein sequences contained in Data Sheet 1. Clade A (orange) and B (yellow) proteins. The number at each node represents the bootstrap percentage value from 1,000 replicates. Smel, Solanum melongena; Sl, Solanum lycopersicum; Stu, Solanum tuberosum.

In addition to the six sequences previously reported (Shetty et al., 2011), four new loci in the eggplant genome were found to encode polyphenol oxidases and named *SmelPPO7-10* (Table 1). Coding sequences retain extensive structural similarities both within *S. melongena* and with homologs in tomato and potato. The CDS of *PPO*s range in size from 1,686 to 2,466 bp; all genes except *SmelPPO3* and *SmelPPO4* are on the negative strand and, like the *PPO* genes of tomato and potato, eggplant *PPO*s do not possess introns. In all the three *Solanum* species, *PPO* genes cluster on chromosome 8 (Figure 1A), with the exception of one orthologous gene (*SmelPPO10* in eggplant, *StuPPO9* in potato, and *SlPPOG* in tomato), mapping on chromosome 2. This suggests that *PPO* genes evolved from tandem duplications and further supports the notion that the structure of this gene family, and possibly its functional specializations, are conserved across Solanaceae species.

**Table 1:** Characteristics of PPO encoding genes and of PPO proteins.

***Locus***	**Gene name**	**Chr**	**Chromosome location**	**ORF length (bp)**	**Strand**	**Size (aa)**	**Protein domains**	**Pfam domains**
SMEL_008g312510.1.01	*Smel_PPO1*	8	97,412,508: 97,414,307	1,800	-	600	PPO1_DWL–DUF_B2219–Tyrosinase	Pfam 12142–Pfam 1243-Pfam 00264
SMEL_008g312500.1.01	*Smel_PPO2*	8	97,401,279: 97,403,066	1,788	-	596	PPO1_DWL–DUF_B2219–Tyrosinase	Pfam 12142–Pfam 1243-Pfam 00264
SMEL_008g312430.1.01	*Smel_PPO3*	8	97,284,426: 97,286,198	1,773	+	591	PPO1_DWL–DUF_B2219–Tyrosinase	Pfam 12142–Pfam 1243-Pfam 00264
SMEL_008g312420.1.01	*Smel_PPO4*	8	97,238,764: 97,239,741	1,734	+	578	PPO1_DWL–DUF_B2219–Tyrosinase	Pfam 12142–Pfam 1243-Pfam 00264
SMEL_008g311990.1.01	*Smel_PPO5*	8	96,314,480: 96,316,243	1,764	-	588	PPO1_DWL–DUF_B2219–Tyrosinase	Pfam 12142–Pfam 1243-Pfam 00264
SMEL_008g312010.1.01	*Smel_PPO6*	8	96,395,550: 96,397,448	1,899	-	633	PPO1_DWL–DUF_B2219–Tyrosinase	Pfam 12142–Pfam 1243-Pfam 00264
SMEL_008g312490.1.01	*Smel_PPO7*	8	97,397,374: 97,399,167	1,794	-	598	PPO1_DWL–DUF_B2219–Tyrosinase	Pfam 12142–Pfam 1243-Pfam 00264
SMEL_008g312460.1.01	*Smel_PPO8*	8	97,349,335: 97,351,020	1,686	-	562	PPO1_DWL–DUF_B2219–Tyrosinase	Pfam 12142–Pfam 1243-Pfam 00264
SMEL_008g312520.1.01	*Smel_PPO9*	8	97,429,811: 97,432,277	2,466	-	822	PPO1_DWL–DUF_B2219–Tyrosinase	Pfam 12142–Pfam 1243-Pfam 00264
SMEL_000g064350.1.01	*Smel_PPO10*	2	982,270: 984,463	2,193	-	731	PPO1_DWL–DUF_B2219–Tyrosinase	Pfam 12142–Pfam 1243-Pfam 00264

*SmelPPO1-9 cluster on chromosome 8, while PPO10, which was initially located on an unanchored scaffold, is probably located on chromosome 2. All PPOs share the same functional domains (PPO1_DWL and Tyrosinase, and a conserved domain of unknown function, DUF_B2219)*.

The *PPO* encoded proteins range in size from 562 to 822 aa (Table 1). All polypeptides possess the same functional domains, namely the central tyrosinase and PPO1_DWL domains, and a C-terminal domain of unknown function (DUF_B2219), characterized by the KFDV conserved motif. In accordance with previous reports (Taranto et al., 2017), we confirmed that in the Solanaceae family two main clusters can be distinguished among PPO proteins (Figure 1B), which correspond to a functional separation between PPOs that are preferentially expressed in roots (tomato *Sl*PPO A-D, potato *Stu*PPO2 and *Stu*PPO4 and eggplant class A proteins, i.e., *Smel*PPO1-3) and PPOs whose expression is associated to defense responses (tomato *Sl*PPO E and F, potato *Stu*PPO1 and eggplant class B proteins, i.e. *Smel*PPO4-6). Among the newly identified proteins, *Smel*PPO7 clusters with class A proteins, *Smel*PPO8 with *Stu*PPO5 and *Smel*PPO9 with *Stu*PPO8. Finally, *Smel*PPO10 clusters with *Stu*PPO9 and *Sl*PPOG.

### Transcriptional Profiling in Response to Wounding

Oxidative browning in eggplant is influenced by multiple factors, including total phenolic content, *PPO* expression and also the way in which the plant integrates environmental stimuli to elicit defense responses (Mishra et al., 2013; Plazas et al., 2013; Docimo et al., 2016). The differential spatial and temporal expression patterns of PPOs *in planta* reflect the functional diversity among the *PPO* gene members. In eggplant, the expression of *SmelPPO1-6* genes was higher in young tissues and declined during plant development in mature and reproductive organs (Shetty et al., 2011). In fruits, *PPO* expression was mainly concentrated in the exocarp and in the areas surrounding the seeds in the mesocarp (Shetty et al., 2011). *PPO* expression is mainly induced by herbivores or by mechanical damage, such as cutting.

The promoters of group B genes (Shetty et al., 2011) are characterized by the presence of several responsive elements for wounding stress and defense response (Thipyapong et al., 1997). The structural similarity of eggplant class B *PPO* genes (*SmelPPO4-5-6*) to wound-induced tomato *SlPPOF* might suggest an analogous pattern of gene regulation (Thipyapong et al., 1997).

**Figure 2:**
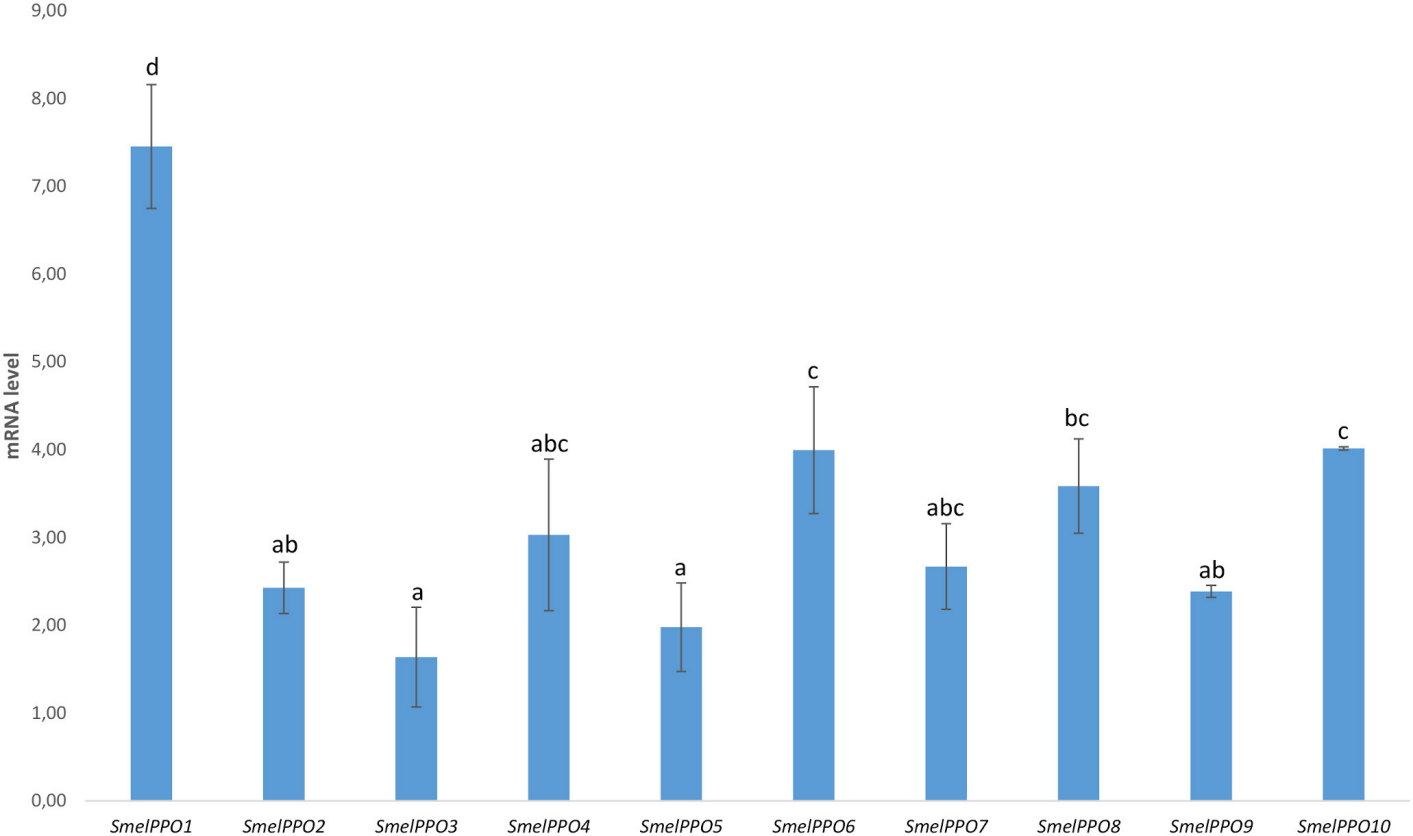
Transcriptional levels of 10 PPO-encoding genes in the Black Beauty variety 30 min after fruit cutting. The values are expressed as relative mRNA abundance at 30 min after cutting compared to time 0 (just after cutting). Eggplant actin and elongation factor genes were used as the reference genes. Data are means of three biological replicates ± SE. Different letters associated with the set of means indicate a significant difference based on Tukey b test (*P* ≤ 0.05).

In our study we analyzed the transcript levels of *PPO* genes in the flesh of full-ripe eggplant berries of the “Black Beauty” variety 30 min after cutting (Figure 2). A strong increase in gene transcription in the flesh was observed for all *PPO*s, and especially for *SmelPPO1* (7.45X), *SmelPPO4* (3.03X), *SmelPPO6* (4.00X), *SmelPPO8* (3.59X), and *SmelPPO10* (4.01X). The simultaneous activation of both A and B classes of *PPO* genes was already observed in the eggplant cultivars AM086 (Docimo et al., 2016) and Arka Shirish (Shetty et al., 2011). Based on this transcriptional profile, we hypothesized that the design of an appropriate editing strategy directed at reducing detrimental oxidative browning in fruit tissues might require simultaneous suppression of several members of this multigene family. In our experiments we targeted class B *PPO* genes (*SmelPPO4, SmelPPO5*, and *SmelPPO6*) through a CRISPR/Cas9 editing strategy. Due to their extremely high level of similarity, it was possible to design a unique gRNA against the tyrosinase domain of all class B genes.

### Plant Regeneration

The development of new genome editing technologies in plant breeding has fostered a growing interest for *in vitro* culture and regeneration protocols, which represent a major bottleneck in the application of these techniques in many plant species of agricultural and industrial interest. Due to the difficulties often encountered in eggplant regeneration, with available protocols being mostly inefficient or highly dependent on the genotype, no examples of genome editing in this species has been reported in literature so far. After the first report of the *Agrobacterium*-mediated transformation of eggplant (Guri and Sink, 1988), several examples of genetic transformation have been proposed using seedling explants like the hypocotyl, epicotyl, and node segments and cotyledon segments, leaf disks or roots (Rotino et al., 2014; Saini and Kaushik, 2019; García-Fortea et al., 2020).

In many plant species, the browning of tissues, which leads to toxicity and necrosis, is one of the major causes of unsuccessful *in vitro* organogenesis and regeneration from explants. Browning is associated with the oxidation of phenolics, whose release is caused by cutting and manipulating explants and calli. This problem is particularly relevant in eggplant, whose tissues are rich in phenolic compounds. Among strategies to avoid browning, the most common include the supplementation of culture media with antioxidant or adsorbent compounds (Abdelwahd et al., 2008; Menin et al., 2013).

We tested different strategies to reduce browning during eggplant tissue culture, including the addition of citric and ascorbic acid and PVP supplementation, and we found out that PVP supplementation exerts a positive effect on shoot regeneration. Among a total of 15 rooted shoots, 10 derived from the PVP-supplemented medium, four from not supplemented medium, and only 1 from the medium supplemented with ascorbic and citric acids. No differences were found in the phenotype of regenerants from different culture conditions. However, in spite of their notably higher number, the emergence of shoots on the PVP-supplemented medium was slower.

Dark treatments are known to increase adventitious shoot formation in cotyledon, leaf and hypocotyl explants in a number of species, including eggplant (Muktadir et al., 2016). However, we did not observe differences between shoots which underwent the 3-days dark treatment and those which did not. For all regenerating conditions, shoots apt for rooting were recovered in as short as 6 weeks (Supplementary Figure 1).

### Screening for Mutations in the T_0_ Generation

**Figure 3:**
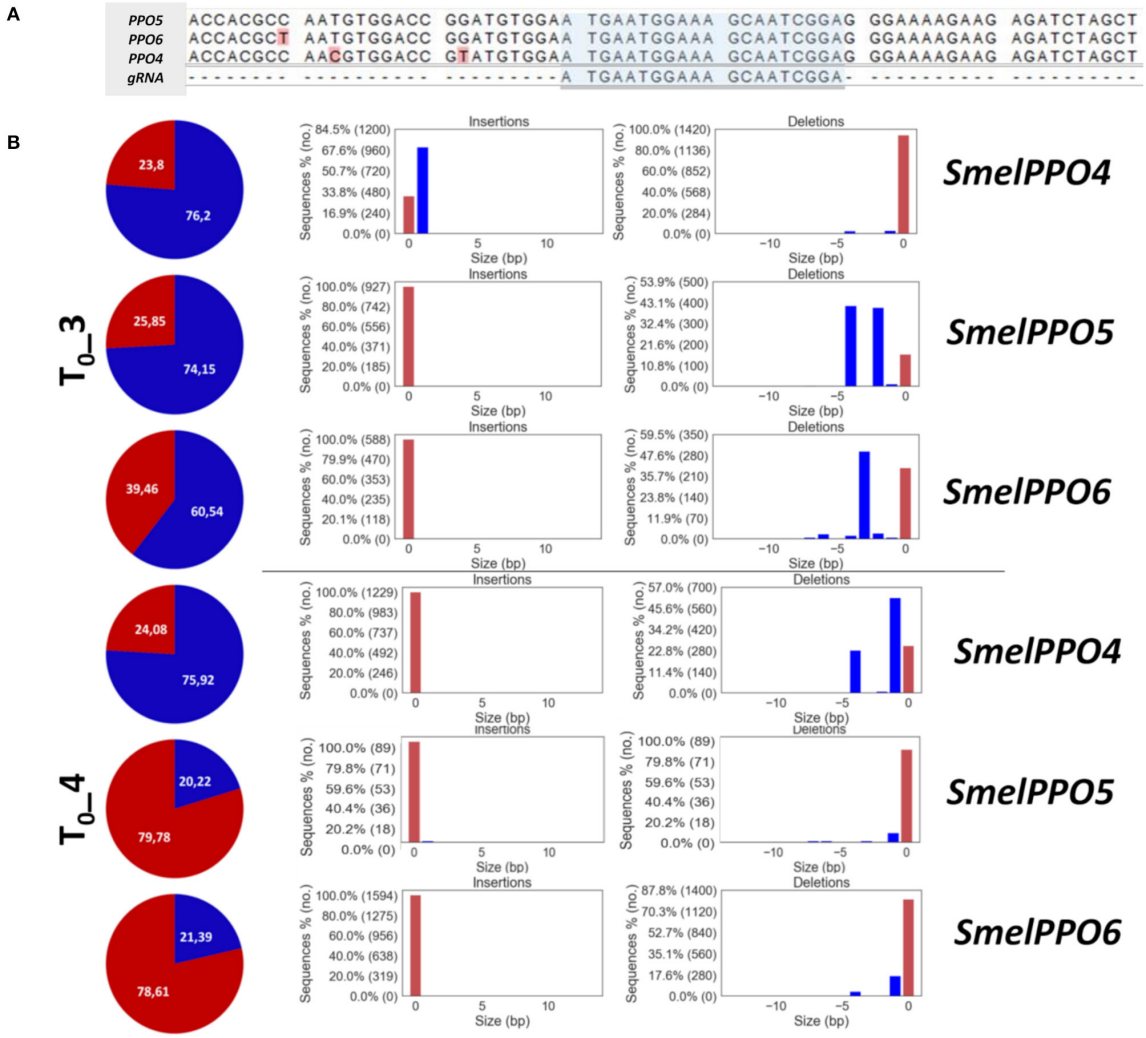
**(A)** Alignment of *SmelPPO4, SmelPPO5 SmelPPO6* with selected g*RNA*. **(B)** Genotyping of targeted gene mutations induced by CRISPR/Cas9 in the T_0_ generation. Quantification of Illumina reads edited at the target locus in T_0__3 and T_0__4. For each line, the percentage of reads carrying mutated (blue) as well as not mutated (red) target sequence is reported together with the pattern and frequency of targeted gene mutations.

CRISPR/Cas9 induced mutagenesis can be employed to induce the simultaneous knockout of multiple targets within a gene family (Karunarathna et al., 2020; Sashidhar et al., 2020). Targeting a conserved gene family poses some challenges regarding the design of the gRNAs as well as the screening of edited genotypes and off-target effects. This can be particularly problematic for *PPO*s, since different members of this gene family, including the ones implicated in defense response, possess distinct activation patterns and specialized metabolic functions. By identifying a conserved region of *SmelPPO4* and *SmelPPO5*, corresponding to the tyrosinase domain, we designed a gRNA targeting both *SmelPPO4* and *SmelPPO5*, as well as *SmelPPO6* (Figure 3A).

After transformation of the CRISPR/Cas9 constructs in eggplant cotyledons and regeneration, 12 eggplant T_0_ individuals (T_0__1-T_0__12) were analyzed (Data Sheet 3). The qPCR analysis using *Cas9* gene-specific primers revealed genomic integration of the construct in nine T_0_ plants, while T_0__1, T_0__6 and T_0__10 did not possess the transgene. In order to detect mutations in *SmelPPO4-5-6*, we employed targeted deep sequencing of genomic DNA, which allowed us to comprehensively assess the editing efficiency and the types of mutations (Data Sheet 3). Among the nine transformed plants, the Illumina amplicon sequencing revealed that simultaneous editing of *SmelPPO4, SmelPPO5, and SmelPPO6* genes occurred in 2 lines (T_0__3 and T_0__4) (Figure 3B). For the remaining lines, T_0__5 and T_0__12 showed editing only at the *SmelPPO5* locus, while T_0__10 at the *SmelPPO4* locus (Data Sheet 3). In most transformants, *SmelPPO5* appears edited to a higher extent than *SmelPPO4* and *SmelPPO6*, with the exceptions of T_0__3, T_0__4 and T_0__10. Notably, in T_0__5 *SmelPPO5* reached an editing efficiency of 50% while the other two loci displayed negligible levels of mutation. Preferential editing of one member of a family sharing the same gRNA recognition sequence might depend on the transcriptional status of the target sequences. Although after wounding the expression of *SmelPPO5* is induced at lower levels than the ones of *SmelPPO4* and *SmelPPO6*, its transcript abundance seems to be generally higher, as suggested by its Ct values. The transcriptional accessibility of this locus might also reflect on its availability for the Cas9 endonuclease. It is interesting to point out that, in T_0__10, *SmelPPO4* was edited with an efficiency of over 70% although the transgene was not integrated, which highlights that it is possible to retrieve non-transgenic plants derived from edited cells in which the editing machinery presumably acted in a “transient” fashion.

T_0__3 showed the greatest editing efficiency for all three loci, i.e., 76% for *SmelPPO4*, 74% for *SmelPPO5* and 60% for *SmelPPO6*. In T_0__4, a high editing efficiency was also detected, i.e., 76% for *SmelPPO4*, 20% for *SmelPPO5* and 21% for *SmelPPO6* (Data Sheet 3 and Figure 3B), although these values are lower than those of T_0__3. The number of plants edited at all loci was low (22%) and editing efficiencies were also significantly below the ones observed in tomato and potato, presumably as a consequence of low levels of expression of Cas9 and gRNAs (Pan et al., 2016). T_0__3 and T_0__4 had chimeric mutations (with at least 3 different alleles) in all targeted loci and retained a proportion of the wild type allele. The wild type copy of the target gene in chimeric plants could thus continue to mutate either in T_0_ or in the following generations if the Cas9 transgene does not segregate. The predominance of this chimeric status in the T_0_ resembles the pattern described in tomato (Pan et al., 2016; Nonaka et al., 2017). Chimerism suggests that gene editing occurred after the emergence of differentiated tissues, leading to a heterogeneous mutation pattern within the same plant. Transgene expression might be influenced by the chromatin status at its insertion locus and, of course, by the choice of promoters. In Arabidopsis, where mutants are obtained through floral dipping and where the expression of Cas9 in the germline is crucial to fix edited alleles, the use of egg cell-specific promoters for Cas9 expression allowed to efficiently obtain non-mosaic T_1_ mutants for multiple target genes (Wang et al., 2015). In plants regenerated through somatic organogenesis, the use of egg-cell and embryo-specific promoters might also help retrieving T_1_ generations with higher levels of homozygous or biallelic mutations (Zheng et al., 2020). Since no previous reports of gene editing dynamics existed for eggplant, and because we predicted the regeneration process to be the limiting factor (both in terms of efficiency and time consumption), we prioritized the use of standard gene editing constructs to maximize Cas9 expression and establish a baseline protocol. Based on this, other variants (e.g., tissue- or species-specific promoters) can be successively factored in to fine-tune the editing outcome.

Previous observations showed that small indels are the predominant mutations introduced in plants by gene editing and that the breakpoint introduced by Cas9 is placed at 3 nucleotides upstream of the PAM (Bortesi et al., 2016; Pan et al., 2016; Andersson et al., 2017). In plants, insertion of one nucleotide or deletion of 1–10 nucleotides are the most common mutations (Pan et al., 2016). The most common mutations in our T_0_ eggplant plantlets were represented by a single nucleotide insertion (+G; T_0__3-*SmelPPO4*) and by a deletion of one (T_0__4-*SmelPPO4/6*; T_0__5-*SmelPPO5*), two (T_0__3-*SmelPPO5*), three (T_0__3-*SmelPPO6*), or four (T_0__4-*SmelPPO*5; T_0__10-*SmelPPO4;* T_0__12-*SmelPPO5*) nucleotides (Data Sheet 3).

### Analysis of Off-Target Mutations

Only few occurrences of low-frequency off-target mutations induced by CRISPR/Cas9 have been reported in plant species so far (Feng et al., 2013; Peterson et al., 2016; Wolt et al., 2016; Hahn and Nekrasov, 2019) contrary to what observed in human cells (Fu et al., 2013). The risk of off-target effects has been reported as comparable to that of somaclonal variation deriving from plant tissue culture itself (Ma et al., 2015). In order to reduce off-target effects, a strategy based on Cas9/sgRNA ribonucleoprotein complexes has been proposed (Hahn and Nekrasov, 2019). Indeed, only through a whole genome resequencing of the edited lines is it possible to exhaustively evaluate the presence of off-target mutations induced by the selected sgRNAs. However, other screening methods make it possible to rule out the occurrence of undesired mutations at selected loci, which is reliable particularly if they correspond to transcriptionally active sequences.

One of the major risks related to targeting conserved regions in a gene family is that putative off-targets are most likely other members of the same family which, in the case of PPOs, are also located in close proximity on the genome. This makes it much more difficult to eliminate potentially undesired off-targets by breeding, than it is for non-linked loci. With respect to our gRNA, four putative off-target sequences were identified: one was an intergenic sequence, while three corresponded to other members of the *PPO* family (*SmelPPO2, SmelPPO3*, and *SmelPPO7*) (Supplementary Table 2).

In order to confirm that our selected T_0_ edited lines (T_0__3 and T_0__4) displayed mutations only in the *SmelPPO4-5-6* loci, we sequenced the candidate off-target loci by applying the same Illumina Amplicon Sequencing Protocol we used for the sequencing of target loci, and which allowed us to get a deep insight into possible non-specific editing activity. The total variation at putative off-target sites was compared between edited and wild type plants (Table 2). We seldom observed only base substitutions consistent with SNPs or sequencing errors and, even considering those, no increase in total variation was observed between wild type and mutants. Our analyses thus demonstrated the lack of off-target effects, confirming the specificity of Cas9-mediated *PPO* gene editing in eggplant. The presence of mismatches in the seed region between our selected sgRNA and the off-target *SmelPPO*s supports the specificity of our results (Hahn and Nekrasov, 2019), since this 3′ terminal region of the target sequence is known to strongly affect recognition by the Cas9 endonuclease.

**Table 2:** Quantification of Illumina reads edited at putative off-target *loci* in T_0_ generation.

**Sample**	**Target**	**Reads**	**Number of mutated reads**	**% of mutated reads**
WT	OT1	17,926	549	3.06
	OT2	17,855	1,354	7.58
	OT3	27,671	10,758	38.88
	OT4	26,977	803	2.98
T_0__3	OT1	24,196	747	3.09
	OT2	21,205	922	4.35
	OT3	30,086	3,203	10.65
	OT4	34,541	822	2.38
T_0__4	OT1	20,621	591	2.87
	OT2	13,231	476	3.60
	OT3	20,282	1,648	8.13
	OT4	274	8	2.92

*For each individual and for each locus the total number of reads is reported, together with the percentage of reads carrying the mutated off-target sequence. The percentage of mutated sequences is reported*.

### Segregation of the Transgene and of Mutated Alleles in the T_1_ and T_2_ Progeny

Due to the early finding that in Arabidopsis many somatic mutations were not efficiently inherited, concerns about the heritability of CRISPR/Cas-induced mutations were initially raised (Feng et al., 2013). However, in all other edited monocot and dicot species, T_1_ generations with high mutation efficiencies have been obtained, demonstrating the heritability of edited alleles (Miao et al., 2013; Gao et al., 2015; Li et al., 2015; Svitashev et al., 2015; Pan et al., 2016). In our case, 14 T_1_ plants of the T_0__4 progeny were examined to investigate the transmission pattern of CRISPR/Cas9-induced mutations. Out of 14 analyzed individuals, 4 presented no detectable amplification of *hCas9* and therefore it is reasonable to deduce that the transgene was segregated (Data Sheet 3).

**Figure 4:**
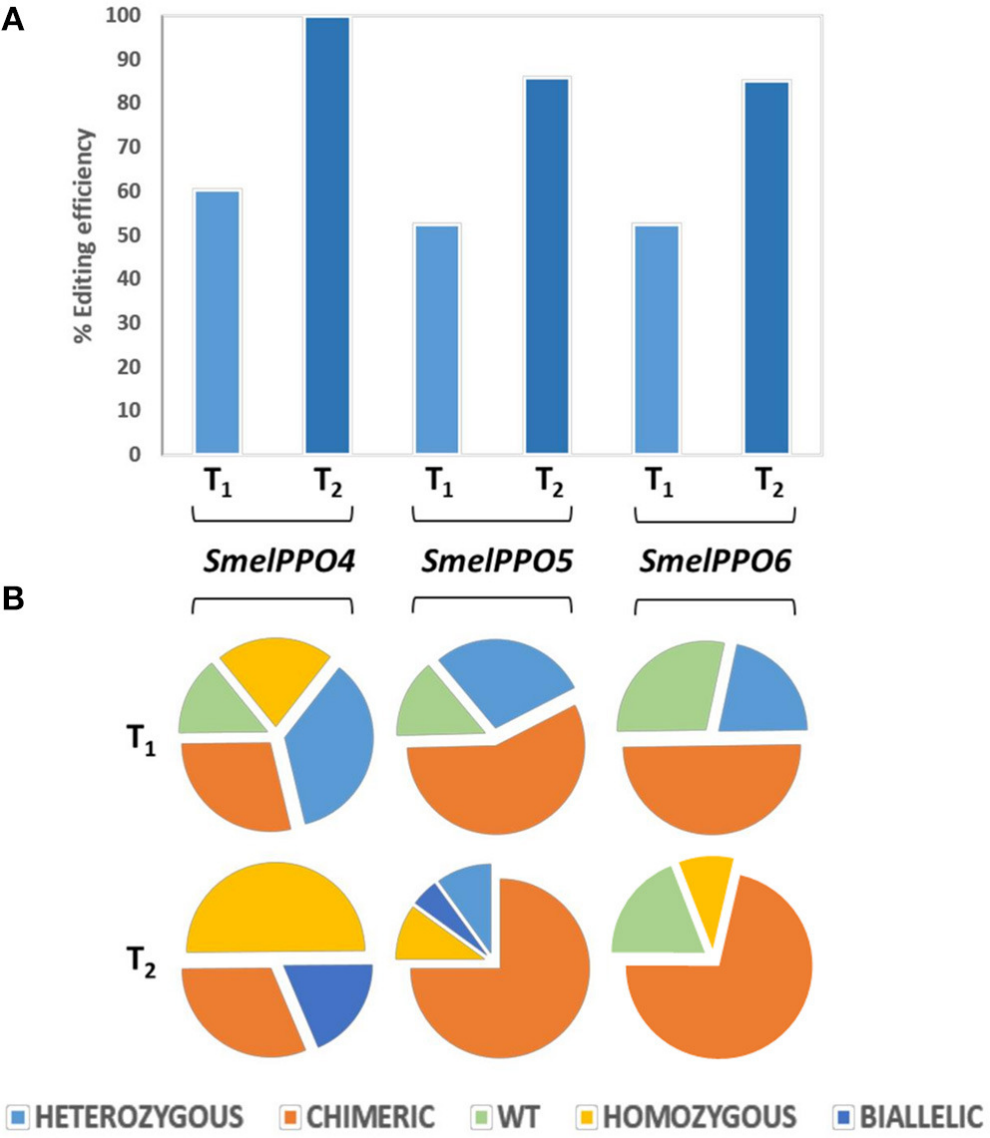
Genotyping of targeted gene mutations induced by CRISPR/Cas9 in the T_1_ and T_2_ generations. **(A)** Mutagenesis frequencies for all three targeted loci in T_1_ and T_2_ progenies. **(B)** Zygosity of targeted gene mutations in T_1_ and T_2_ populations.

In order to detect the mutation efficiency and patterns at different sites in *SmelPPO4-5-6* genes, we employed targeted deep sequencing. The average editing efficiency was 60% for *SmelPPO4*, 52% for *SmelPPO5*, and 52% for *SmelPPO6* (Figure 4A). Focusing on the *SmelPPO4* locus, five were heterozygous mutants, four chimeric, three homozygous and two WT. At the *SmelPPO5* locus, four were homozygous mutants, eight chimeric and two WT. At the *SmelPPO6* locus, three were homozygous mutants, seven chimeric and four WT (Figure 4B; Data Sheet 3).

The most common mutation at the *SmelPPO4* locus was a single nucleotide deletion, followed by a 4 nucleotide deletion. At the *SmelPPO5* locus different mutations were present:−2/-1/-4/-3. The segregation pattern at the *SmelPPO6* locus (which was less mutated in T_0_) was less predictable and a number of new mutations (-2/-3/-7) were found in the T_1_ lines. The highest editing efficiency was highlighted for T_1__4_10: 85% for *SmelPPO4*, 90% for *SmelPPO5* and 90.7% for *SmelPPO6* (Data Sheet 3).

To further investigate the genetic stability of the targeted mutations we screened the T_2_ plants derived from selfing T_1__4_8, T_1__4_9 and T_1__4_10 (Data Sheet 3). The presence of a transgene in most of the analyzed T_2_ plants (19/21) suggested that more than one copy of the transgene was inserted in those T_0_ regenerants, which explains that Cas9 can still be active in all T_2_ plants.

**Figure 5:**
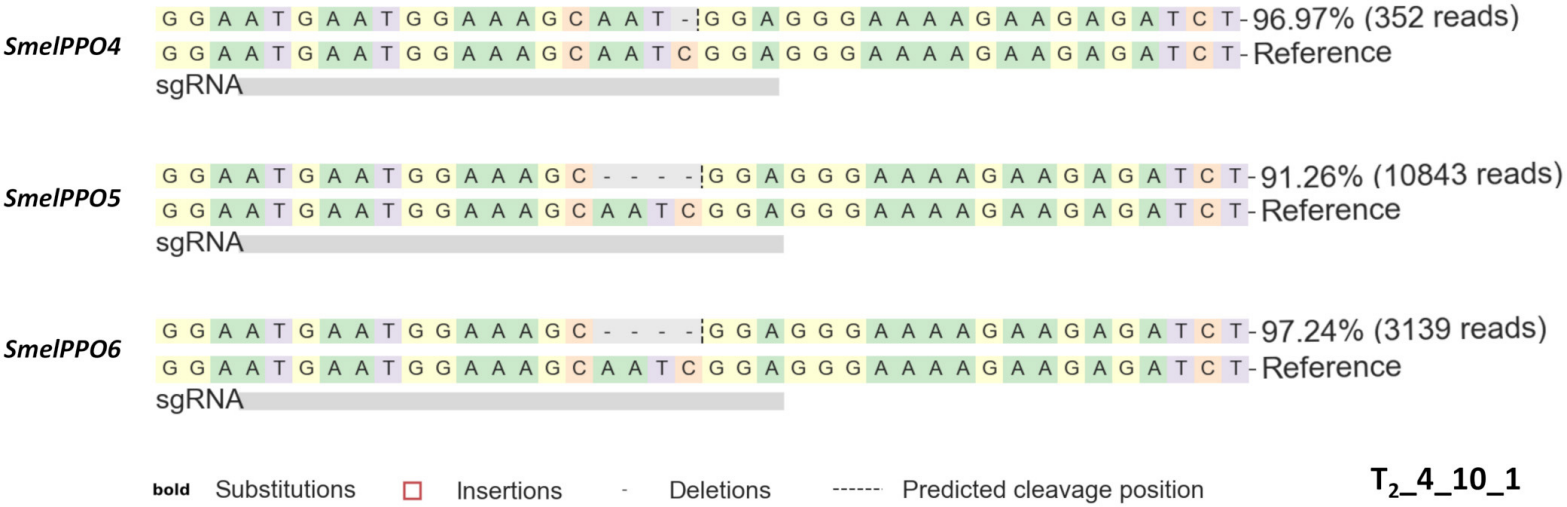
Genotyping of targeted gene mutations induced by CRISPR/Cas9 in the T_2__4_10_1 plant. The dashed lines represent nucleotide deletions. The reported number represents the frequency and the number of reads carrying mutated (edited) target sequence.

Compared to the T_1_ generation, the mutagenesis frequency (99% for *SmelPPO4*, 85% for *SmelPPO5* and 85% for *SmelPPO6*) as well as the overall proportion of homozygous, biallelic and chimeric assets increased (Figure 4). As expected, all 7 T2 progeny of T_1__4_11 were homozygous at the *SmelPPO4* locus, indicating that the mutations in the homozygotes were stably passed to the next generation in a Mendelian fashion. As previously observed in other species (Pan et al., 2016), the segregation patterns of the T_1_ chimera lines were less predictable and a number of new mutants were obtained due to the probable continued Cas9 activity. Interestingly, T_2__4_10_1 showed homozygous mutations for *SmelPPO4* (-1/-1), *SmelPPO5* (-4/-4) and *SmelPPO6* (-4/-4) (Figure 5).

It has been previously demonstrated how off-target effects can be further exacerbated in the T_2_ progeny as compared to T_0_ and T_1_ (Zhang et al., 2018). Targeted deep sequencing at putative off-target loci once again demonstrated the lack of significant mutated off-targets in our T_2_ progeny, confirming the specificity of Cas9-mediated *PPO* gene editing in eggplant (Data Sheet 3). We observed only base substitutions consistent with SNPs or sequencing errors, with similar frequencies to those observed in the T_0_, which did not represent an increase in total variation between wild type and mutant lines.

### Enzymatic Browning and PPO Activity Analysis in Eggplant Berries

**Figure 6:**
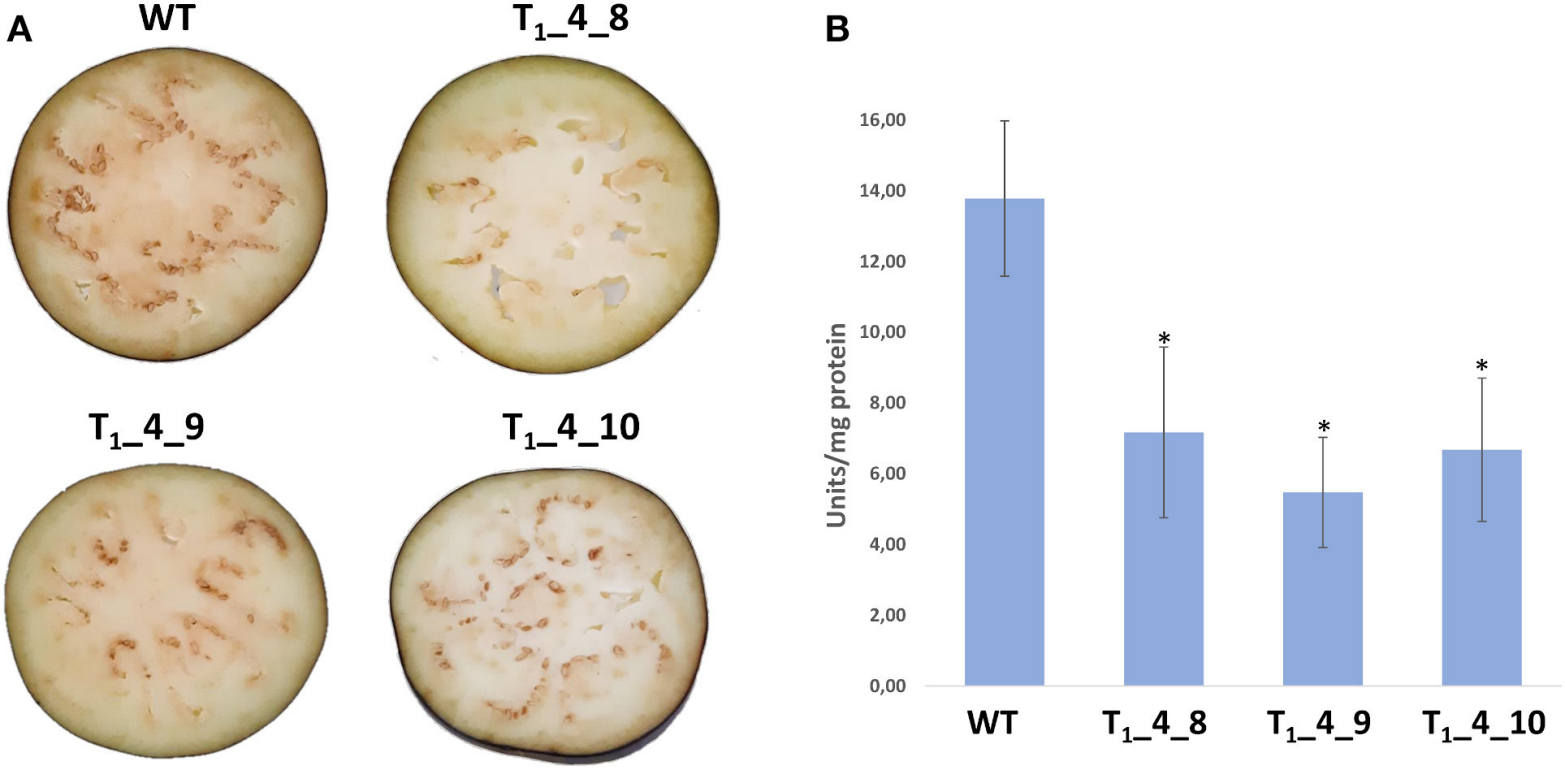
Phenotypical and biochemical changes associated with postcut browning. **(A)** Cut fruits of Wild Type, T_1__4_8, T_1__4_9 and T_1__4_10 showing post-cut browning 30 min after cutting. **(B)** Polyphenoloxidase (PPO) activity in fruits of Wild Type, T_1__4_8, T_1__4_9 and T_1__4_10. Data are means of eight biological replicates ± SD. Asterisks indicate a significant difference based on Tukey's HSD test (*P* ≤ 0.05).

We hypothesized that the CRISPR/Cas-mediated knock out of *PPO*s would result in a lowered enzymatic browning, due to the reduced PPO activity. Selected T_1_ lines (T_1__4_8, T_1__4_9 and T_1__4_10) carrying mutations in *SmelPPO4-5*-*6* genes were subjected to phenotypic analysis of enzymatic browning and PPO activity in berries. The lines were grown in a greenhouse and no growth alteration or changes in berry size/weight were observed during plant development when compared to wild type, as previously observed in potato (Llorente et al., 2011). The berries were cut and exposed to air for browning induction. After 30 min, the typical brown discoloration due to phenolic oxidation was detected and it was clearly more evident in wild type plants in comparison to edited lines (Figure 6A). The average PPO activity of T_1__4_8, T_1__4_9 and T_1__4_10 lines was also found to be reduced by 48, 61, and 52%, respectively, compared to the wild type (Figure 6B). By comparing the T_2_ edited lines with wild type a reduction of PPO activity as well as of browning discoloration upon cutting was highlighted (Supplementary Figure 2).

Several studies applied RNA silencing technologies to down-regulate the expression of *PPO* genes in potato tubers (Bachem et al., 1994; Rommens et al., 2006; Llorente et al., 2011; Chi et al., 2014). In this species, by using the amiRNA technology (Chi et al., 2014), a reduction in PPO activity of 15–95% was obtained and it was more marked when *StuPPO1* to *4* were simultaneously suppressed. Furthermore, in a more recent study in potato, CRISPR/Cas mutants for the four alleles of the *StuPPO2* gene (which is considered the major contributor to the PPO protein content) displayed a reduction up to 69 and 73% in the PPO activity and enzymatic browning, respectively (González et al., 2020)

We can hypothesize that the partial reduction of PPO activity in eggplant, comparable to the one observed in potato mutants, might be enhanced through the knockout not only of class B PPOs (*SmelPPO4-5-6*), but even of class A PPOs (*SmelPPO1* and *SmelPPO3*). However, this approach could provoke downside effects, due to the involvement of the *PPO* multigene family in important cell functions (Jukanti and Bhatt, 2015).

## Conclusions

We have established a successful protocol for gene editing in eggplant, adding to the list of Solanaceae species for which CRISPR/Cas9 represents an alluring option for the introduction of specific traits through a biotechnological approach. Our system, based on the use of one guide RNA directed simultaneously at three members of the *PPO* gene family, demonstrated to be specific for the target genes, without detectable off-target effects on other members of the same gene family.

Upon cutting, edited T_1_ and T_2_ eggplant fruits showed a reduction of the typical brown coloration due to phenolic oxidation. Through our approach it will be possible to develop eggplant varieties that maintain their antioxidant and nutritional properties during harvest and post-harvest procedures, without reducing the content in phenolics, which are beneficial for human health.

Phenolics provide a substrate to oxidative reactions catalyzed by PPOs that, consuming oxygen and producing fungitoxic quinones, play a role in making the medium unfavorable to the further development of pathogens (Taranto et al., 2017); however, contrasting results are reported in literature, as *PPO* silenced lines of potato were found to acquire higher resistance to *P. infestans* (Llorente et al., 2014). Our future studies will be thus focused in assessing the relationship between PPO knock-out and pathogen response in our mutant eggplant lines.

## Data Availability Statement

The original contributions presented in the study are included in the article/Supplementary Materials, further inquiries can be directed to the corresponding author/s.

## Author Contributions

AMo, SG, and CC: conceptualization. AMa, SG, AMo, and AA: data curation. AMa, SG, AMo, AA, DV, and AMM: investigation. AMo and CC: supervision. AMa, SG, and AMo: writing—original draft preparation. SG, AMo, JP, DO, AG, SL, and CC: writing—review and editing preparation. All authors contributed to the article and approved the submitted version.

## Conflict of Interest

The authors declare that the research was conducted in the absence of any commercial or financial relationships that could be construed as a potential conflict of interest.

## References

[B1] Abdelwahd R. Hakam N. Labhilili M. Udupa S. M. (2008). Use of an adsorbent and antioxidants to reduce the effects of leached phenolics in *in vitro* plantlet regeneration of faba bean. African J. Biotechnol. 7, 997–1002.

[B2] Andersson M. Turesson H. Nicolia A. Fält A. S. Samuelsson M. Hofvander P. (2017). Efficient targeted multiallelic mutagenesis in tetraploid potato (Solanum tuberosum) by transient CRISPR-Cas9 expression in protoplasts. Plant Cell Rep. 36, 117–128. 10.1007/s00299-016-2062-3 27699473 PMC5206254

[B3] Bachem C. W. B. Speckmann G. J. Van der Linde Piet C. G. Verheggen F. T. M. Hunt M. D. Steffens J. C. (1994). Antisense expression of polyphenol oxidase genes inhibits enzymatic browning in potato tubers. Bio/Technology. 12, 1101–1105. 10.1038/nbt1194-1101

[B4] Barchi L. Pietrella M. Venturini L. Minio A. Toppino L. Acquadro A.. (2019). A chromosome-anchored eggplant genome sequence reveals key events in Solanaceae evolution. Sci. Rep. 9:11769. 10.1038/s41598-019-47985-w 31409808 PMC6692341

[B5] Bellés J. M. Garro R. Pallás V. Fayos J. Rodrigo I. Conejero V. (2006). Accumulation of gentisic acid as associated with systemic infections but not with the hypersensitive response in plant-pathogen interactions. Planta. 223, 500–511. 10.1007/s00425-005-0109-8 16331468

[B6] Bolger A. M. Lohse M. Usadel B. (2014). Trimmomatic: a flexible trimmer for Illumina sequence data. Bioinformatics 30, 2114–2120. 10.1093/bioinformatics/btu170 24695404 PMC4103590

[B7] Bortesi L. Zhu C. Zischewski J. Perez L. Bassié L. Nadi R.. (2016). Patterns of CRISPR/Cas9 activity in plants, animals and microbes. Plant Biotechnol. J. 14, 2203–2216. 10.1111/pbi.12634 27614091 PMC5103219

[B8] Bradford M. M. (1976). A rapid and sensitive method for the quantitation of microgram quantities of protein utilizing the principle of protein-dye binding. Anal. Biochem. 72, 248–254. 10.1016/0003-2697(76)90527-3 942051

[B9] Chi M. Bhagwat B. Lane W. D. Tang G. Su Y. Sun R.. (2014). Reduced polyphenol oxidase gene expression and enzymatic browning in potato (Solanum tuberosum L.) with artificial microRNAs. BMC Plant Biol. 14:62. 10.1186/1471-2229-14-62 24618103 PMC4007649

[B10] Clement K. Rees H. Canver M. C. Gehrke J. M. Farouni R. Hsu J. Y.. (2019). CRISPResso2 provides accurate and rapid genome editing sequence analysis. Nat. Biotechnol. 37, 224–226. 10.1038/s41587-019-0032-3 30809026 PMC6533916

[B11] Coetzer C. Corsini D. Love S. Pavek J. Turner N. (2001). Control of enzymatic browning in potato (solanum tuberosum L.) by sense and antisense RNA from tomato polyphenol oxidase. J. Agric. Food Chem. 49, 652–657. 10.1021/jf001217f 11262007

[B12] Cong L. Zhang F. (2015). Genome engineering using CRISPR-Cas9 system. Methods Mol. Biol. 1239:197–217. 10.1007/978-1-4939-1862-1_10 25408407

[B13] Docimo T. Francese G. De Palma M. Mennella D. Toppino L. Lo Scalzo R.. (2016). Insights in the fruit flesh browning mechanisms in Solanum melongena genetic lines with opposite postcut behavior. J. Agric. Food Chem. 64, 4675–4685. 10.1021/acs.jafc.6b00662 27198496

[B14] Doudna J. A. Charpentier E. (2014). The new frontier of genome engineering with CRISPR-Cas9. Science 346:1258096. 10.1126/science.1258096 25430774

[B15] Doyle J. Doyle J. (1987). A rapid isolation procedure for small amounts of leaf tissue. Phytochem. Bull. 19, 11–15.

[B16] Feng Z. Zhang B. Ding W. Liu X. Yang D. L. Wei P.. (2013). Efficient genome editing in plants using a CRISPR/Cas system. Cell Res. 23, 1229–1232. 10.1038/cr.2013.114 23958582 PMC3790235

[B17] Fu Y. Foden J. A. Khayter C. Maeder M. L. Reyon D. Joung J. K.. (2013). High-frequency off-target mutagenesis induced by CRISPR-Cas nucleases in human cells. Nat. Biotechnol. 31, 822–826. 10.1038/nbt.2623 23792628 PMC3773023

[B18] Gao J. Wang G. Ma S. Xie X. Wu X. Zhang X.. (2015). CRISPR/Cas9-mediated targeted mutagenesis in Nicotiana tabacum. Plant Mol. Biol. 87, 99–110. 10.1007/s11103-014-0263-0 25344637

[B19] García-Fortea E. Lluch-Ruiz A. Pineda-Chaza B. J. García-Pérez A. Bracho-Gil J. P. Plazas M.. (2020). A highly efficient organogenesis protocol based on zeatin riboside for in vitro regeneration of eggplant. BMC Plant Biol. 20:6. 10.1186/s12870-019-2215-y 31906864 PMC6945591

[B20] González M. N. Massa G. A. Andersson M. Turesson H. Olsson N. Fält A. S.. (2020). Reduced enzymatic browning in potato tubers by specific editing of a polyphenol oxidase gene via ribonucleoprotein complexes delivery of the CRISPR/Cas9 system. Front. Plant Sci. 10:1649. 10.3389/fpls.2019.01649 31998338 PMC6962139

[B21] Guri A. Sink K. C. (1988). Agrobacterium transformation of eggplant. J. Plant Physiol. 133, 52–55. 10.1016/S0176-1617(88)80083-X

[B22] Hahn F. Nekrasov V. (2019). CRISPR/Cas precision: do we need to worry about off-targeting in plants? Plant Cell Rep. 38, 437–441. 10.1007/s00299-018-2355-9 30426198 PMC6469637

[B23] Jukanti A. K. Bhatt R. (2015). Eggplant (Solanum melongena L.) polyphenol oxidase multi-gene family: a phylogenetic evaluation. 3 Biotech. 5, 93–99. 10.1007/s13205-014-0195-z 28324357 PMC4327750

[B24] Karunarathna N. L. Wang H. Harloff H. J. Jiang L. Jung C. (2020). Elevating seed oil content in a polyploid crop by induced mutations in SEED FATTY ACID REDUCER genes. Plant Biotechnol. J. 18, 2251–2266. 10.1111/pbi.13381 32216029 PMC7589255

[B25] Kaushik P. Gramazio P. Vilanova S. Raigón M. D. Prohens J. Plazas M. (2017). Phenolics content, fruit flesh colour and browning in cultivated eggplant, wild relatives and interspecific hybrids and implications for fruit quality breeding. Food Res. Int. 102, 392–401. 10.1016/j.foodres.2017.09.028 29195964

[B26] Li L. Steffens J. C. (2002). Overexpression of polyphenol oxidase in transgenic tomato plants results in enhanced bacterial disease resistance. Planta 215, 239–247. 10.1007/s00425-002-0750-4 12029473

[B27] Li Z. Liu Z. Bin X.ing A. Moon B. P. Koellhoffer J. P. Huang L.. (2015). Cas9-guide RNA directed genome editing in soybean. Plant Physiol. 169, 960–970. 10.1104/pp.15.00783 26294043 PMC4587461

[B28] Llorente B. Alonso G. D. Bravo-Almonacid F. Rodríguez V. López M. G. Carrari F.. (2011). Safety assessment of nonbrowning potatoes: opening the discussion about the relevance of substantial equivalence on next generation biotech crops. Plant Biotechnol. J. 9, 136–150. 10.1111/j.1467-7652.2010.00534.x 20497372

[B29] Llorente B. López M. G. Carrari F. Asís R. Di Paola Naranjo R. D. Flawi,á M. M. (2014). Downregulation of polyphenol oxidase in potato tubers redirects phenylpropanoid metabolism enhancing chlorogenate content and late blight resistance. Mol. Breed. 34, 2049–2063. 10.1007/s11032-014-0162-8

[B30] Ma X. Zhang Q. Zhu Q. Liu W. Chen Y. Qiu R.. (2015). A Robust CRISPR/Cas9 system for convenient, high-efficiency multiplex genome editing in monocot and dicot plants. Mol. Plant. 8, 1274–1284. 10.1016/j.molp.2015.04.007 25917172

[B31] Mahanil S. Attajarusit J. Stout M. J. Thipyapong P. (2008). Overexpression of tomato polyphenol oxidase increases resistance to common cutworm. Plant Sci. Volume 174, 456–466. 10.1016/j.plantsci.2008.01.006

[B32] Menin B. Moglia A. Comino C. Hakkert J. C. Lanteri S. Beekwilder J. (2013). In vitro callus-induction in globe artichoke (Cynara cardunculus L. var. scolymus) as a system for the production of caffeoylquinic acids. J. Hortic. Sci. Biotechnol. 88, 537–542. 10.1080/14620316.2013.11513003

[B33] Mennella G. Lo Scalzo R. Fibiani M. DAlessandro A. Francese G. Toppino L.. (2012). Chemical and bioactive quality traits during fruit ripening in eggplant (S. melongena L.) and allied species. J. Agric. Food Chem. 60:11821–11831. 10.1021/jf3037424 23134376

[B34] Miao J. Guo D. Zhang J. Huang Q. Qin G. Zhang X.. (2013). Targeted mutagenesis in rice using CRISPR-Cas system. Cell Res. 23, 1233–1236. 10.1038/cr.2013.123 23999856 PMC3790239

[B35] Mishra B. B. Gautam S. Sharma A. (2013). Free phenolics and polyphenol oxidase (PPO): The factors affecting post-cut browning in eggplant (Solanum melongena). Food Chem. 139, 105–114. 10.1016/j.foodchem.2013.01.074 23561085

[B36] Muktadir M. A. Habib M. A. Khaleque Mian M. A. Yousuf Akhond M. A. (2016). Regeneration efficiency based on genotype, culture condition and growth regulators of eggplant (*Solanum melongena* L.). Agric. Nat. Resour. 50, 38–42. 10.1016/j.anres.2014.10.001

[B37] Naveed M. Hejazi V. Abbas M. Kamboh A. A. Khan G. J. Shumzaid M.. (2018). Chlorogenic acid (CGA): a pharmacological review and call for further research. Biomed. Pharmacother. 97, 67–74. 10.1016/j.biopha.2017.10.064 29080460

[B38] Nonaka S. Arai C. Takayama M. Matsukura C. Ezura H. (2017). Efficient increase of Γ-aminobutyric acid (GABA) content in tomato fruits by targeted mutagenesis. Sci. Rep. 7:7057. 10.1038/s41598-017-06400-y 28765632 PMC5539196

[B39] Pan C. Ye L. Qin L. Liu X. He Y. Wang J.. (2016). CRISPR/Cas9-mediated efficient and heritable targeted mutagenesis in tomato plants in the first and later generations. Sci. Rep. 6:24765. 10.1038/srep24765 27097775 PMC4838866

[B40] Peterson B. A. Haak D. C. Nishimura M. T. Teixeira P. J. P. L. James S. R. Dangl J. L.. (2016). Genome-wide assessment of efficiency and specificity in crispr/cas9 mediated multiple site targeting in arabidopsis. PLoS ONE. 13:e0162169. 10.1371/journal.pone.0162169 27622539 PMC5021288

[B41] Plazas M. López-Gresa M. P. Vilanova S. Torres C. Hurtado M. Gramazio P.. (2013). Diversity and relationships in key traits for functional and apparent quality in a collection of eggplant: fruit phenolics content, antioxidant activity, polyphenol oxidase activity, and browning. J. Agric. Food Chem. 61, 8871–8879. 10.1021/jf402429k 23972229

[B42] Prohens J. Rodríguez-Burruezo A. Raigón M. D. Nuez F. (2007). Total phenolic concentration and browning susceptibility in a collection of different varietal types and hybrids of eggplant: implications for breeding for higher nutritional quality and reduced browning. J. Am. Soc. Hortic. Sci. 132, 638–646. 10.21273/JASHS.132.5.638

[B43] Rommens C. M. Ye J. Richael C. Swords K. (2006). Improving potato storage and processing characteristics through all-native DNA transformation. J. Agric. Food Chem. 54, 9882–9887. 10.1021/jf062477l 17177515

[B44] Rotino G. L. Sala T. Toppino L. (2014). “Eggplant,” in Alien Gene Transfer in Crop. Plants 2, 381–409. 10.1007/978-1-4614-9572-7_16

[B45] Saini D. K. Kaushik P. (2019). Visiting eggplant from a biotechnological perspective: a review. Sci. Hortic. (Amsterdam). 253, 327–340. 10.1016/j.scienta.2019.04.042

[B46] Sashidhar N. Harloff H. J. Potgieter L. Jung C. (2020). Gene editing of three BnITPK genes in tetraploid oilseed rape leads to significant reduction of phytic acid in seeds. Plant Biotechnol. J. 18, 2241–2250. 10.1111/pbi.13380 32191373 PMC7589381

[B47] Shetty S. M. Chandrashekar A. Venkatesh Y. P. (2011). Eggplant polyphenol oxidase multigene family: cloning, phylogeny, expression analyses and immunolocalization in response to wounding. Phytochemistry 72, 2275–2287. 10.1016/j.phytochem.2011.08.028 21945722

[B48] Svitashev S. Young J. K. Schwartz C. Gao H. Falco S. C. Cigan A. M. (2015). Targeted mutagenesis, precise gene editing, and site-specific gene insertion in maize using Cas9 and guide RNA. Plant Physiol. 169, 931–945. 10.1104/pp.15.00793 26269544 PMC4587463

[B49] Taranto F. Pasqualone A. Mangini G. Tripodi P. Miazzi M. M. Pavan S.. (2017). Polyphenol oxidases in crops: biochemical, physiological and genetic aspects. Int. J. Mol. Sci. 18:377. 10.3390/ijms18020377 28208645 PMC5343912

[B50] Thipyapong P. Hunt M. D. Steffens J. C. (2004). Antisense downregulation of polyphenol oxidase results in enhanced disease susceptibility. Planta 220, 105–117. 10.1007/s00425-004-1330-6 15300439

[B51] Thipyapong P. Joel D. M. Steffens J. C. (1997). Differential expression and turnover of the tomato polyphenol oxidase gene family during vegetative and reproductive development. Plant Physiol. 13, 707–718. 10.1104/pp.113.3.707 PMC158188 12223637

[B52] Van Eck J. (2018). Genome editing and plant transformation of solanaceous food crops. Curr. Opin. Biotechnol. 49, 35–41. 10.1016/j.copbio.2017.07.012 28800419

[B53] Wang Z. P. Xing H. L. Dong L. Zhang H. Y. Han C. Y. Wang X. C.. (2015). Egg cell-specific promoter-controlled CRISPR/Cas9 efficiently generates homozygous mutants for multiple target genes in Arabidopsis in a single generation. Genome Biol. 16:144. 10.1186/s13059-015-0715-0 26193878 PMC4507317

[B54] Wolt J. D. Wang K. Yang B. (2016). The Regulatory Status of Genome-edited Crops. Plant Biotechnol. J. 14, 510–518. 10.1111/pbi.12444 26251102 PMC5042095

[B55] Zhang Q. Xing H. L. Wang Z. P. Zhang H. Y. Yang F. Wang X. C.. (2018). Potential high-frequency off-target mutagenesis induced by CRISPR/Cas9 in Arabidopsis and its prevention. Plant Mol. Biol. 96, 445–456. 10.1007/s11103-018-0709-x 29476306 PMC5978904

[B56] Zheng N. Li T. Dittman J. D. Su J. Li R. Gassmann W.. (2020). CRISPR/Cas9-Based Gene Editing Using Egg Cell-Specific Promoters in Arabidopsis and Soybean. Front. Plant Sci. 11:800. 10.3389/fpls.2020.00800 32612620 PMC7309964

