# Supplementary material for: Simultaneous CRISPR/Cas9 Editing of Three PPO Genes Reduces Fruit Flesh Browning in Solanum melongena L
Source: Front Plant Sci. 2020 Dec 3;11:607161. doi: 10.3389/fpls.2020.607161 (PMC7744776; doi:10.3389/fpls.2020.607161)
Supplement: Supplementary file 1 [file Table_1.DOCX]

**Supplementary Table 1:** List of primers used for qPCR analysis

| **Locus** | **Primer Forward** | **Primer Reverse** |
| --- | --- | --- |
| *SmelPPO1* | TCTGATGGTCCTGAAGTACCC | ATGAGCAGGCTGACGGATAC |
| *SmelPPO2* | ACAGAGTTGGCGAGAATACTGAC | TTTGGTACCAGAGTCACCGC |
| *SmelPPO3* | AGGTCTCGGTGCTGCTAATC | TAGACGGCATAGGAGGTGGG |
| *SmelPPO4* | TGTGGTAAATGACACAACGGGC | CTCCTCATCCACCGCATGAG |
| *SmelPPO5* | AACTGAACTCCAAACAATGACG | CCATTCCTGGACTTGGATCG |
| *SmelPPO6* | AGTGTTCCATACTACAAGTTCCCTC | CCAAGAGGGTCAAAAGGGTCT |
| *SmelPPO7* | TGCGGATAAGAATTTGAATGCGGAT | GCTCTGTGAACGTGTGGCAAG |
| *SmelPPO8* | TGCCCTTCCCAATTCTTCGG | TACGACGTGGCACCATTACC |
| *SmelPPO9* | ATGCACCTTGTCCTCAGCTG | ACCAACCCATCGATGCACAG |
| *SmelPPO10* | ACCTTCGCGATGCCATTTTG | TGCCTCCTATCTCTGAGCCG |
| *SmelACTIN* | ACCACAGCTGAGCGAGAAAT | GACCATCGGGAAGCTCATAG |
| *SmelEF* | ACCAGCATCACCATTCTTCA | ACTGCCATACTTCCCACATT |
