# Supplementary material for: Simultaneous CRISPR/Cas9 Editing of Three PPO Genes Reduces Fruit Flesh Browning in Solanum melongena L
Source: Front Plant Sci. 2020 Dec 3;11:607161. doi: 10.3389/fpls.2020.607161 (PMC7744776; doi:10.3389/fpls.2020.607161)
Supplement: Supplementary file 2 [file Table_2.DOCX]

**Supplementary Table 2:** Characteristics of putative off-target sites for the gRNAs directed at *SmelPPO4-5-6*. Mismatches are in lowercase and bulges are represented by dashes.

| **Off-target** | **Chromosome** | **Sequence** | **Annotation** |
| --- | --- | --- | --- |
| *SmelPPO4-5-6* OT1 | SMEL3Ch08 | TGAATGG_AAAcaAATCGGA-GGGA | *SmelPPO3* |
| *SmelPPO4-5-6* OT2 | SMEL3Ch08 | cGAATGG_AAgGCAATaGGA-GGGA | *SmelPPO7* |
| *SmelPPO4-5-6* OT3 | SMEL3Ch08 | cGAATGG_AAAGCgATaGGA-GGGA | *SmelPPO2* |
| *SmelPPO4-5-6* OT4 | SMEL3Ch06 | TGAtTGG_AAActAATCGGA-TGGT | none |
