# Supplementary material for: Simultaneous CRISPR/Cas9 Editing of Three PPO Genes Reduces Fruit Flesh Browning in Solanum melongena L
Source: Front Plant Sci. 2020 Dec 3;11:607161. doi: 10.3389/fpls.2020.607161 (PMC7744776; doi:10.3389/fpls.2020.607161)
Supplement: Supplementary file 3 [file Table_3.DOCX]

**Supplementary Table 3:** Primers used for target and off-target sequencing. (A) Primers used for Cas9 screening. (B) Primers used for the first PCR amplification of *SmelPPO4* and *SmelPPO6*. (C) Primers used for the Amplicon Illumina amplification of *SmelPPO4*, *SmelPPO5* and *SmelPPO5*, and for potential off-target loci for the *PPO4-5-6* gRNAs. In bold is the universal Illumina adapter, while the 3' terminal part of the primer is specific for each locus.

**(A)**

| **Primer name** | **Primer sequence** |
| --- | --- |
| *Cas9*_For | CTATCCTCAGGCGGCAAGAG |
| *Cas9*_Rev | AGTCATCCACGCGAATCTGG |

**(B)**

| **Primer name** | **Primer sequence** |
| --- | --- |
| *SmelPPO4*_outer_For | AGTACTCTCCCATTATGCACCA |
| *SmelPPO4*_outer_Rev | TCCACAAGATCAATCACTGCAT |
| *SmelPPO6* _outer_For | TGCACCAACAAATCTCTCTCT |
| *SmelPPO6* _outer_Rev | ACAATCCATAAGCACGATCTCC |

**(C)**

| **Primer name** | **Primer sequence** |
| --- | --- |
| *SmelPPO4*_ For | **TCGTCGGCAGCGTCAGATGTGTATAAGAGACAGTCACACTCCAGTCCACAT** |
| *SmelPPO4*_ Rev | **GTCTCGTGGGCTCGGAGATGTGTATAAGAGACAGAATTCCGAGTTCAACCAATC** |
| *SmelPPO6*_ For | **TCGTCGGCAGCGTCAGATGTGTATAAGAGACAGTCACAATGCGGTCCACAA** |
| *SmelPPO6*_ Rev | **GTCTCGTGGGCTCGGAGATGTGTATAAGAGACAGAATTCCGAGTTCAACCAATC** |
| *SmelPPO5*_For | **TCGTCGGCAGCGTCAGATGTGTATAAGAGACAG**CTCTGGGAACCGATCCAAGT |
| *SmelPPO5*_Rev | **GTCTCGTGGGCTCGGAGATGTGTATAAGAGACAG**GATTCCGAGTTCAACCAATC |
| OT1_For | T**CGTCGGCAGCGTCAGATGTGTATAAGAGACAG**GGAGTGCAGGGAACCATT |
| OT1_Rev | **GTCTCGTGGGCTCGGAGATGTGTATAAGAGACAG**GAATTCTGAGTCCAGCCAATC |
| OT2_For | **TCGTCGGCAGCGTCAGATGTGTATAAGAGACAG**GGGAACCGTTGAAGTCAT |
| OT2_Rev | **GTCTCGTGGGCTCGGAGATGTGTATAAGAGACAG**GAACTCCGAGTTCAACCAA |
| OT3_For | **TCGTCGGCAGCGTCAGATGTGTATAAGAGACAG**AACCATTGAAGTCATCCCT |
| OT3_Rev | **GTCTCGTGGGCTCGGAGATGTGTATAAGAGACAG**GAGTTCAACCAATCTTTATGTG |
| OT4_For | **TCGTCGGCAGCGTCAGATGTGTATAAGAGACAG**TTGCGGGATAAAGAAGTTG |
