# Supplementary material for: Simultaneous CRISPR/Cas9 Editing of Three PPO Genes Reduces Fruit Flesh Browning in Solanum melongena L
Source: Front Plant Sci. 2020 Dec 3;11:607161. doi: 10.3389/fpls.2020.607161 (PMC7744776; doi:10.3389/fpls.2020.607161)
Supplement: Supplementary file 4 [file Table_4.DOCX]

**Supplementary Table 4:**

1. Parameters used for CRISPResso2 analysis <https://crispresso.pinellolab.partners.org/submission> of editing events.

| Minimum homology for alignment to an amplicon: | 80% |
| --- | --- |
| Center of the quantification window (relative to 3' end of the provided sgRNA): | -3 |
| Quantification window size (bp): | 10 |
| Minimum average read quality (phred33 scale): | >10 |
| Minimum single bp quality (phred33 scale): | >10 |
| Replace bases with N that have a quality lower than (phred33 scale) | <10 |
| Exclude bp from the left side of the amplicon sequence for the quantification of the mutations: | 15 |
| Exclude bp from the right side of the amplicon sequence for the quantification of the mutations | 15 |
| Trimming adapter | Nextera PE |
| Only mutation representing >5% of total aligned reads |  |

1. Target amplicon sequences and gRNA.

| **TARGET** | **Amplicon sequence** | **gRNA *SmelPPO4-5-6*** |
| --- | --- | --- |
| *SmelPPO4* | TCACACTCCAGTCCACATCTGGACCGGTGACTCACCTAGACAACCAAACGGCGAGGACATGGGTAATTTCTACTCAGCCGGTCTAGACCCGGTTTTCTATTGCCACCACGCCAACGTGGACCGTATGTGGAATGAATGGAAAGCAATCGGAGGGAAAAGAAGAGATCTAGCTGATAAAGATTGGTTGAACTCGGAATT | ATGAATGGAAAGCAATCGGA |
| *SmelPPO5* | CTCTGGGAACCGATCCAAGTCCAGGAATGGGCACTATCGAAAACATTCCTCATAATCCGGTCCACATCTGGACCGGTGACTCACCTAGACAACCAAACGGCGAGGACATGGGTAATTTCTACTCAGCCGGTCTAGACCCGGTTTTCTATTGCCACCACGCCAATGTGGACCGGATGTGGAATGAATGGAAAGCAATCGGAGGGAAAAGAAGAGATCTAGCTGATAAAGATTGGTTGAACTCGGAATC |  |
| *SmelPPO6* | GCGGTCCACAACTGGACCGGTGACCAACCTAGACAACCAAACGGCGAGCACATGGGTACTTTCTACTCAGCCGGTCTAGACCCGGTTTTCTATTCCCACCACGCTAATGTGGACCGGATGTGGAATGAATGGAAAGCAATCGGAGGGAAAAGAAGAGATCTAGCTGATAAAGATTGGTTGAACTCGGAATT |  |

1. Putative off-target amplicon sequences and gRNAs.

| **OFF TARGET** | **Amplicon sequence** | **gRNA** |
| --- | --- | --- |
| OT1 | GGAGTGCAGGGAACCATTGAAAAAATCCCTCACACTCCTGTACACATTTGGGTCGGTACAAAGAAAGATTCAATTCTACCTAATGGTAAAAAGTCATACGGTGAGGATATGGGTAATTTCTACTCAGCTGCTTTGGACCCGGTTTTCTATTGCCATCACAGCAATGTGGACCGAATGTGGAATGAATGGAAACAAATCGGAGGGAAAAGAAGGGATCTCTCGCAAAAAGATTGGCTGGACTCAGAATTC | ATGAATGGAAACAAATCGGA |
| OT2 | GAACTCCGAGTTCAACCAATCTTTATGTGAGATATCCCTTCTTTTCCCTCCTATTGCCTTCCATTCGTTCCACATCCGGTCCACATTGCTGTGATGGCAGTAGAAAACCGGGTCCAAACCAGCTGAGTAGAAATTACCCATGTCCTCGCCGTGTGACCTTGAACCATCAGGCAAAATCGAACCTGCCCTTGTACCAGTCCAAACGTGGACAGGACCGTGAGGGATGACTTCAACGGTTCCC | TCCTATTGCCTTCCATTCG |
| OT3 | GAGTTCAACCAATCTTTATGTGAGATATCCCTTCTTTTCCCTCCTATCGCTTTCCATTCGCTCCACATCCGGTCCACGTTACTGTGGTGGCAAAAGAAAACCGGGTCCAAACCAGCTGAGTAGAAATGACCCATGTTCTCACCGTGTGACGTTCTACCATTAGGCAAGGTTGTACCTGGCACTGTACCAGTCCAAACGTGGACAGGACCGTGAGGGATGACTTCAATGGTT | TCCTATCGCTTTCCATTCG |
| OT4 | TTGCGGGATAAAGAAGTTGTTTTCAATAGATTCTTAGCTCAAGGAGATTATTCTCAAATAAAGGTTTGAAAGAAAAGCAAGATTAAAAGGTCAAGTTAAAATATTGAATATTAAAGATAATCAAAGTAAAAGAAACATTGATTGGATTGATGGTTTGTGAGCATTGAATCTTGAAAGATGTGATTGGAAACTAATCGGATGGTTGATAGGCCGAAAGGCTTGATTGTGATGGATTGATAAGAGTTC | GTGATTGGAAACTAATCGGA |
