# Supplementary material for: Simultaneous CRISPR/Cas9 Editing of Three PPO Genes Reduces Fruit Flesh Browning in Solanum melongena L
Source: Front Plant Sci. 2020 Dec 3;11:607161. doi: 10.3389/fpls.2020.607161 (PMC7744776; doi:10.3389/fpls.2020.607161)
Supplement: Supplementary file 5 [file Data_Sheet_1.DOCX]

**Supplementary File 1.** PPO protein sequences used for tree construction. *Solanum melongena (Smel)*, *Solanum lycopersicum (Sl)*, *Solanum tuberosum (Stu)*.

**>Stu08g017870.1.1_StuPPO1**

MSSSSTTTLPLCTNKSLSSSFTTNNSSFLSKPSQLFLHGRRNQSFKVSCNVNNNVGEHEKNLDAVDRRNVLLGLGGLYGAANLAPLASASPIPPPDLKSCGVAHVKEGVDVLYSCCPPVPDDIDSVPYYKFPPMTKLRIRPPAHAADEEYVAKYQLATSRMRELDKDSFDPLGFKQQANIHCAYCNGAYKVGGKELQVHFSWLFFPFHRWYLYFYERILGSLINDPTFALPYWNWDHPKGMRIPPMFDREGSSLYDDKRNQNHRNGTIIDLGHFGKEVDTPQLQIMTNNLTLMYRQMVTNAPCPSQFFGAAYPLGTKPSPGMGTIENIPHTPVHIWTGDTPRQKNGENMGNFYSAGLDPIFYCHHANVDRMWDEWKLIGGKRRDLSNKDWLNSEFFFYDENRNPYRVKVRDSLDSKKMGFSYAPMPTPWRNFKPIRKTTAGKVNTASIAPVTKVFPLAKLDRAISFSITRPASSRTTQEKNEQEEILTFNKIAYDDTQYVRFDVFLNVDKTVNADELDKAEFAGSYTSLPHVHGNNTNHVTSVIFKLAITELLEDNGLEDEDTIAVTLVPKVGGEGVSIESVEIKLEDC*

**>Stu08g019390.1.1_StuPPO2**

MASLCNSSNTSLKTPFTSSSTSLSSTPKPSSTFHPWKTYQMFKVSCKVTNNNGDQNQNVETNSVDRRNVLLGLGGLYGVANAIPLAASASPTPPPDLSSCSKATINETTVVPYSCCAPKPDDMEKVPYYKFPSMTKLRVRQPAHEANEEYIAKYNLAVSKMRDLDKTQPLNPIGFKQQANIHCAYCNGAYRIGGKELQVHNSWLFFPFHRWYLYFYERIVGKLIDDPTFALPYWNWDHPKGMRFPAMYDREGTSLFDVTRDQSHRNGAVIDIGFFGNEVETTQLQLMSNNLTLMYRQMVTNAPCPRMFFGGPYDLGSNVELPGTIENIPHGPVHIWSGTVRGSTLPNGAISNGENMGHFYSAGLDPVFFCHHSNVDRMWSEWKATGGKRTDITHKDWLNSEFFFYDENENPYRVKVRDCLDTKKMGYDYKPMATPWRNFKPLTKASAGKKVNTSSIPPVSQVFPLAKLDKAISFSINRPTSSRTQQEKNAQEEMLTFSSIRYDNRGYIRFDVFLNVDNNVNANELDKAEFAGSYTSLPHVHRAGETNHIATVDFQLAITELLEDIGLEDEETIAVTLVPKRGGEGISIEGATISLADC*

**>Stu08g019410.1.1_StuPPO3**

MASVCNSSSTTTTLKTPFISSNTSLSSTPKPSQLFLHGKRNQMFKVSCKVTNNNGDQNVETNSVDRRNVLLGLGGLYGVANAIPLAASAAPTPPPDLKTCGKATISDGPLVGYTCCPPPMPTNFDNIPYYKFPSMTKLRIRSPAHAVDEEYIAKYNLAISRMKDLDKTEPLNPLGFKQQANIHCAYCNGAYVFGDKVLQVHNSWLFFPFHRWYLYFYERILGKLIDDPTFALPYWNWDHPKGMRLPPMFDREGTSIYDERRNQQVRNGTVMDLGSFGDKVETTQLQLMSNNLTLMYRQMVTNAPCPLLFFGAPYVLGNNVEAPGTIENIPHIPVHIWAGTVRGSTFPNGDTSYGEDMGNFYSAGLDSVFYCHHGNVDRMWNEWKAIGGKRRDLSEKDWLNSEFFFYDENKKPYRVKVRDCLDAKKMGYDYAPMPTPWRNFKPKTKVSAGKVNTSSLPPVNEVFPLAKMDKVISFSINRPASSRTQQEKNEQEEMLTFDNIKYDNRGYIRFDVFLNVDNNVNANELDKVEFAGSYTSLPHVHRVGENDHTATVTFQLAITELLEDIGLEDEETIAVTLVPKKGGEGISIENVEIKLLDC*

**>Stu08g019380.1.1_StuPPO4**

MASLCNSSSTTLKTPFTSSSTSLSSTPKPSQLFLHGKRNKTFKVSCKVTNNNGDQNQNVETNSVDRRNVLLGLGGLYGVANAIPLAASASPTPPPDLSSCSKATINETTEVPYSCCAPTPKDMTKVPYYKFPSMTKLRIRPPAHALDEAYIAKYNLAISRMKDLDKTQPTNPIGFKQQANIHCAYCNGAYVIDGKVLQVHNSWLFFPFHRWYLYFYERILGSLINDPTFALPYWNWDHPKGMRFPPMFDIPGTALYDERRGEQIHNGTIIDLGSFGDQVQTTQLQLMTNNLTLMYRQLVTNAPCPLMFFGGPYTLGSDVESPGTVEVIPHSPVHIWAGTRRGSILPDGKTSNGEDMGHFYSAGLDPVFYCHHSNVDRMWKEWKAIGGKRTDIQNKDWLNSEFFFYDESGNPFKVKVRDCLDTKKMGYDYEPMPTPWRNFKPKTKASAGKKVNTSSIPPVSQVFPLAKLDKAISFSINRPASSRTQQEKNAQEEMLTFNEIKYDNRDYIRFDVFLNVDNNVNANELDKAEFAGSYTSLPHVHRVGDTNHTATATLQLAITELLEDIGLEDDETIAVTLVPKKGGISIGGVEIKLADC*

**>Stu08g019310.1.1_StuPPO5**

MSKLRKRPVAQDVTKEYIAKYQLETKRMKELDKDDPRSFMQQANIHCAYCNGAYKFGDEILQVHQSWLFFPFHRWYLFFYDRILGKLIDDPTFALPYWNWDHPKGMRLPPMFDRANTPLYDARREDMGNFYSAALDPVFYCHHANVDRMWKVWKGLCGKRRDIIDPDWLNSEFFFYDENKNPYRVRVGDCLDTKKMGYDYAPAAIPWINCRPTRKGREGKVVVTNIKPANKVFPIANLNKPISFSINRPTTSRSQKDKDEKEEVLIFKGLKYDTNKYIRFDVFLNEDEDMNTDELDKVEFAGSYVNLPHVHAHNQKMDSDEMFQLGITELLEDIELEDDDTITVTVVPKTGGDVISIQSVAIELLDG*

**>Stu08g017890.1.1_StuPPO6**

MKRCKAKTLIPAMASSILPLCTTNIPSSFSNNTNSSFLSKPSQLFLHGRRSQSFKVSCNYSEHDKNNLHDDAVDRRNVLLGLGGLYGAANLAPLATAAPSPPPDLKTCSTATVTPGGPAVDYSCCPPPIPTDMSTIPYYKFPPMNKPRIRSPAHLADEEYIAKYNLAITRMKNLDKTEPLNPLGFKQQANIHCAYCNSAYRIGGKELQVHNSWLFFPFHRWYIYFYERILGSLIDDPTFALPYWNWDNPKGMRLPPMFDREGTSLYDERRNPQVRNRTVMDLGTFGKYKVDTTELLLINNNLILMYRQMVTNAPCPLLFFGAPYVLGTNIEAPGTIENIPHITVHIWTGTVRDSTFPNGDTSYGEDMGNFYSAGLDPVFYCHHGNVDRMWNEWKALGGRRTDLTQPDWLNSEFFFYDENKNPYLVKVQDCLDMKKMGYDYAPSPTPWLNFKPNKKYSTGILDTSLVPPARKIFPIFKLDVNTSFSLDRPASSRTQVEKDNKEEILTFSFIKYDNREYRRFDVYLNADKNVNIEDLNQKEYAGSYTSLPHTHKPGDDDHVSSATFQLAITEVLEENGLENEEKIMVTLVPRKGGEGLAIGCVEIMLVDC*

**>Stu08g019300.1.1_StuPPO7**

MASSCSINSMCLSLGEQSSKTLIITTPSSFFAKPSRRSQNFHVSCNNNANNGDEHDKNVARRNVLLGLGGLYGASNLAPLASASPIPAPNLKSCGKATKTGSTKEVGYSCCPLTPDDWNNIPYYKFPPISKLRKSPVAQDVTEEYIAKYQLATQRMKDLDKKNPCSFMQQANIHCAYCNGAYKFGDEVLQRILGKLIDDPTFALPYWNWDHPKGMRLPPMFHRETTSLYDARRNPHVRNGTIIDFSSSTDEVYTDVKKTVTNNLTVMYHQMITNAACPLQFFGARYVLGNNNMNDRGTIENSPHTPVHIWTGTVKGTVLGDGKPSFGEDMGNFYSAALDPVFYSHHANVDRMWNIWKGLGWKKKDITDTDWLNSEFFFYDEHERPYRVRVGDCLDTKMGYDYAPADIPWINCRPTRKGREGKVDLTNIEPANKVFPIANLNKPISFCINRPTTSRSQKDKDEKEEVLIFKGLKYDTSIYISFDVFLNEDEDVNTNELDKVEFAGSYVNLPHVHAHNKRMDYGETFQLDIIELLEDIGLEDDDTITVTVVPKKGGEVISIQSVAIEFLEG*

**>Stu08g019420.1.1_StuPPO8**

MASNFLLTSCTTISSSPSKIFVRPKRIDNFKVNCEIKNSNTINNDDNEGKSFPGKLDRRNVLLGLGGLYGASNLIGVTNEPFALGAPVPPPDFSTCSTASLPDGSQVPFSCCPPLPKDLTNIPTYKLPNVSKVKIRPAAHNVTQEYITKYNTAIQKMKSLDKDDPLSFIQQANIHCAYCNSGYKELGFPGVPLQVHFSWLFFPFHRWYLYFFERILGSLIGDDTFALPFWNYDSQVGMQFPSLYNDVNSSLYDPNRNQNHFPPNVIDLGFTTIDLDASDQQKINNNLTMMYRQMLTNAPCPQLFFGNPIRGGEQPIRGMGTIENVPHNSVHRWVGNPNNKFRENMGTFYSAARDPIFYAHHANVDRMWTIWKTLGGNRRDFNDRDWLDSAFLFYDENRTLVKVTVQDCINNEKLGYKYENVPIPWKNYKPVPRKQKLKKNPKNVKPSTEIFPSTLKKTLSFSIKRPKISRTQQDKDIEEELLVFNNMTFDENEYIRFDVFINEDEGVKSKVLDRTEYVGSFANLPHVHAAGNNTGSSSSGTPAVMSLAISEILEDLGLEDEEEIVVVVVPKFGGKEITIASVEIDTLACAN

**>Stu02g021500.1.1_StuPPO9**

MFMNTSQTSKKMSSIPLPTTNTLSSSTTTTFSNLHSSPFFAKTSKISSIRKHNVHRNFQVSCKTIDDNSHEHNNSPIDISKKNDSLNNMIDRRNVLLGLGGLYGASTLVGGLPFALAAPVNGPDVTKCGAADLPPGAAPVNCCPPMSGNIIDFQLPSSTALRTRPAAHSVDSAYIEKFNRGIQLMKQLPDDDPRSFKQQANVHCAYCDGAYDQLGFPNSELQVHSSWIFLPFHRCYLYFFERILGSLINDPTFAMPFWNWDHPDGMRMPGLYTNSTSSLYDRLRDRRHQPPTMVDLDFNGTDPNISNAQQTSQNLTNMYRQMVSLGRTPETFLGDPYRAGGVPGGAGSLENMAHGAVHVWTGDRTQANFENMGDFYSAARDPIFYAHHSNIDRLWTVWKTLGGRRQDFTDPDFLNTSFLFYDEKAQMVRIRVRDVLDSSKLGYVYQNVTNPWINSRPTPRVSRALSSVRRLVEAKAADDNSNVMNFPRPKEIFPTKLDHVIKVMVKRPNKKKRNKKEKNEKEEILIVEGLEVESDVFVKFDVLINDEDETLVSPDNAEFVGSFVNVPHHSHGKGEKNSKRKTKLKLAITELLEDLDAENDENVLVTFVPKNGSGAVKIGGVKIVLED*

**>Sl08g074680.3.1_PPO_A**

MYSPMNLHPKLAIMASLCSNSSSTSLKTPFTSSTTCLSSTPKASQLFLHGKRNKTFKVSCKVTNTNGNQDETNSVDRRNVLLGLGGLYGVANAIPLAASAAPTPPPDLSSCNKPKINATTEVPYFCCAPKPDDMSKVPYYKFPSVTKLRIRPPAHALDEAYIAKYNLAISRMKDLDKTQPDNPIGFKQQANIHCAYCNGGYSIDGKVLQVHNSWLFFPFHRWYLYFYERILGSLIDDPTFGLPFWNWDHPKGMRFPPMFDVPGTALYDERRGDQIHNGNFIDLGSFGDQVETTQLQLMTNNLTLMYRQLVTNSPCPLMFFGGPYTLGSTVEAAGTVENIPHSPVHIWVGTRRGSVLPDGKISNGEDMGNFYSAGLDPLFYCHHSNVDRMWNEWKATGGKRTDIQNKDWLNSEFFFYDENGNPFKVRVRDCLDTKKMGYDYQPTATPWRNFKPKTKASAGKVNTGSIPPESQVFPLAKLDKAISFSINRPASSRTQQEKNAQEEVLTFNAIKYDNRDYIRFDVFLNVDNNVNANELDKAEFAGSYTSLPHVHRVGDPKHTATATLRLAITELLEDIGLEDEDTIAVTLVPKKGDISIGGVEIKLADC

**>Sl08g074683.1.1_PPO_B**

MASVVCNSSSSTTTTTLKTPFTSLGSTPKPSQLFLHGKRNKTFKVSCKVINNNGNQDETNSVDRRNVLLGLGGLYGVANAIPLAASATPIPSPDLKTCGRATISDGPLVPYSCCPPPMPTNFDTIPYYKFPSMTKLRIRTPAHAVDEEYIAKYNLAISRMRDLDKTEPLNPLGFKQQANIHCAYCNGAYIIGGKELQVHNSWLFFPFHRWYLYFYERILGKLIDDPTFALPYWNWDHPKGMRLPPMFDREGSSLYDERRNQQVRNGTVLDLGSFGDKVETTQLQLMSNNLTLMYRQMVTNAPCPLLFFGAPYVLGNNVEAPGTIETIPHIPVHIWAGTVRGSKFPNGDVSYGEDMGNFYSAGLDPVFYCHHGNVDRMWNEWKAIGGKRRDISEKDWLNSEFFFYDEHKNPYRVKVRDCLDTKKMGYDYAPMPTPWRNFKPKSKASVGKVNTSTLPPANEVFPLAKMDKTISFAINRPASSRTQQEKNEQEEMLTFNNIRYDNRGYIRFDVFLNVDNNVNANELDKAEFAGSYTSLPHVHRAGENDHIAKVNFQLAITELLEDIGLEDEDTIAVTLVPKKGGEGISIENVEIKLVDC

**>Sl08g074682.1.1_PPO_D**

MASLCSNSSTTSLKTPFTSLGSTPKPCQLFLHGKRNKAFKVSCKVTNTNGNQDETNSVDRRNVLLGLGGLYGVANAIPLAASAAPTPPPDLSSCSIARIDENQVVSYSCCAPKPDDMEKVPYYKFPSMTKLRVRQPAHEADEEYIAKYNLAVSKMRDLDKTQPLNPIGFKQQANIHCAYCNGAYRIGGKELQVHNSWLFFPFHRWYLYFYERIVGKLIDDPTFALPYWNWDHPKGMRFPAMYDREGTFLFDETRDQSHRNGTVIDLGFFGNEVETTQLQMMSNNLTLMYRQMVTNAPCPRMFFGGPYDLGTNVELPGTIENIPHGPVHIWSGTVRGSTLPNGAISNGENMGHFYSAGLDPVFFCHHSNVDRMWSEWKATGGKRTDITHKDWLNSEFFFYDENENPYRVKVRDCLDTKKMGYDYKPMATPWRNFKPLTKASAGKVNTSSIPPVSQAFPLAKLDKAVSFSINRPTSSRTPQEKNAQEEMLTFNSIRYDNRGYIRFDVFLNVDNNVNANELDKAEFAGSYTSLPHVHRAGETNHIATVDFQLAITELLEDIGLEDEDTIAVTLVPKRGGEGISIENATISLADC

**>Sl08g074620.3.1_PPO_E**

MSSSSSITTTLPLCTNKSLSSSFTTTNSSLLSKPSQLFLHGRRNQSFKVSCNANNVDKNPDAVDRRNVLLGLGGLYGAANLAPLATAAPIPPPDLKSCGTAHVKEGVDVIYSCCPPVPDDIDSVPYYKFPSMTKLRIRPPAHAADEEYVAKYQLATSRMRELDKDPFDPLGFKQQANIHCAYCNGAYKVGGKELQVHFSWLFFPFHRWYLYFYERILGSLINDPTFALPYWNWDHPKGMRIPPMFDREGSSLYDEKRNQNHRNGTIIDLGHFGKEVDTPQLQIMTNNLTLMYRQMVTNAPCPSQFFGAAYPLGSEPSPGQGTIENIPHTPVHIWTGDKPRQKNGEDMGNFYSAGLDPIFYCHHANVDRMWNEWKLIGGKRRDLTDKDWLNSEFFFYDENRNPYRVKVRDCLDSKKMGFDYAPMPTPWRNFKPIRKSSSGKVNTASIAPVSKVFPLAKLDRAISFSITRPASSRTTQEKNEQEEILTFNKISYDDRNYVRFDVFLNVDKTVNADELDKAEFAGSYTSLPHVHGSNTNHVTSVTFKLAITELLEDIGLEDEDTIAVTLVPKAGGEEVSIESVEIKLEDC

**>Sl08g074630.2.1_PPO_F**

MSSSTPNTLPLLSTNKSLSSPFTNNHSTFLSKPSQPFLHGRRCQSFKVSCNVGEHDKNLDAVDRRNVLLGLGGFYGAANLAPLASAAPIPPPDLKSCGVAHIDDKGTEVSYSCCPPVPDDIDSVPYYKFPPMTKLRIRPPAHAADEEYVAKYQLATSRMRELDKDPFDPLGFKQQANIHCAYCNGAYKIGGKELQVHFSWLFFPFHRWYLYFYERILGSLINDPTFALPYWNWDHPKGMRIPPMFDREGSSLYDEKRNQNHRNGTIIDLGHFGKDVETPQLQIMTNNLTLMYRQMVTNAPCPSQFFGAAYPLGSDPEPGMGTIENIPHTPVHIWTGDSPRQGHGEDMGNFYSAGLDPLFYCHHANVDRMWNEWKLIGGKRRDLSNKDWLNSEFFFYDENRNPYRVKVRDCLDSKKMGFDYAPMPTPWRNFKPIRRSSSGKVNTASIAPVSKVFPLAKLDRAISFSITRPASSRTTQEKNEQEEILTFNKMAYDDTKYVRFDVFLNVDKTVNAEELDKAEFAGSYTSLPHVHGNNDNHVKDVTFTLAITELLEDIGLEDEDTIAVTLVPKVGGEGVSIESVEIKLEDC

**>Sl02g078650.2.1_PPO_G**

MFMNTPQTSKVMSSILFPTTNTLSSSTTNTFSNLHSSPFFAKTSKISSIRKHNVHRNFQVSCKTIDDNNHEHNSPVDISKKNDSSNNMIDRRNVLLGLGGLYGASTLVGGLPFVLAAPVNGPDVTKCGAADLPPGAELVNCCPPMSGSIIDFQLPSSSTPLRTRPAAHSVDSAYIEKFNRAIQLMKQLPDDDPRSFKQQANIHCAYCDGAYDQLGFPNSELQVHSSWIFLPFHRCYLYFFERILGSLINDPTFAMPFWNWDHPDGMRMPGLYTNPTSSLYDRLRDRRHQPPTMVDLDFNGTDPNISTAQQTSQNLTNMYRQMVSLGRTPETFLGDPYRAGGVPGGAGSLENMAHGAVHVWTGDRTQANFENMGDFYSAARDPIFYAHHSNIDRLWTVWKTLGGRRQDFTDPDFLNTSFLFYDEKAQMVRIRVRDVLDSSKLGYVYQNVRNQWINSRPTPRVSRALSSVRRLVEARAADDNNNNIMNFPRPKEIFPTKLDHVIKVMVKRPNKKKRNKKEKNEREEILIVEGLEVESDVFVKFDVLINDEDETLISPDNAEFAGSFVNVPHHSHGKGEKNSKRKTKLKLAITELLEDLDAENDDNVLVTFVPKNGSGAVKIGGVKIVLED*

**>Smel_008g312510.1.01_1 PPO1**

MASVCNTSTATLKSSFIPSPNSLGSTPKPSQLFLHGKRNQAFKVSCKVTNNNGDQNQNVVDTNSVDRRNVLLGLGGLYGVANAIPLAASATPIPAPNAPSCGTATISDGPEVPYTCCPPGMPEDIEKIPYYKFPSATKLRIRQPAHAVDEELIAKYNLAISKMRELDTTDHFSPLAFKQQANIHCAYCNGAYKIGGKELQVHNSWLFFPFHRWYLYFYERILGKLIDDPTFALPYWNWDHPKGMRLPPIFDRQGTALYDERRSTQVRNGTVMDLGSFGDKVQTTQLQLMSNNLTLMYRQMVTNAPCPLLFFGAPYVLGNNVEAPGTVEVIPHIPVHIWVGTARGSKFPDGSTSYGEDMGNFYSAGLDPVFYCHHSNVDRMWNEWKQIGGKRRDISQRDWLNSEFFFFDENKNPYRVRVRDCLDTKTMGYDYAPMPTPWRNFKPKTKASSGKANTSAFPPASQVFPLAKMDKVITFSIKRPASSRTQQEKNEKEEMLTFNNIKYDNREYVRFDVFLNVDNNVNANELDKAEFAGSYTSLPHVHRASQTDHVATATLQLAITELLEDIGLEDEDTIAVTLVPKKGGEGISIEGVEISLADC*

**>Smel_008g312500.1.PPO2**

MASLCNSSNTTLKTPFTSLGSTPKPSQLFLHGKRKQTFKVSCKVSNNNGDQNQNEVEKNSVDRRNVLLGLGGMYGAANFAPLAASAAPTPPPDLSSCSIAKITETEEVSYSCCAPTPDDLNKIPYYKFPSMTKLRIRQPAHAADEEYIAKYNLAISRMKHLDTTEPLNPIGFKQQANIHCAYCNGAYKIGDKVLQVHNSWLFFPFHRWYLYFYERILGSIIDDPTFALPYWNWDHPKGMRMPAMFDREGTALYDQVRNQSHRNGRVMDLGSFGDEVQTTELQLMSNNLTLMYRQMVTNAPCPRMFFGAPYVLGNNVEAPGTIEVIPHGPVHVWTGTVPGTTLPNGRTSHGENMGHFYSAGLDPVFFCHHSNVDRMWSEWKAIGGKRRDISHKDWLNSEFFFYDENGDPFRVKVRDCLDTKKMGYDYAPMPTPWRNFKPITKASVGKVDTSSLPPVSQVFPLAKLDKAISFSINRPASSRTQQEKNEQEEMLTFNNIKYDNRNYVRFDVFLNVDSNVNADELDKAEFAGSYTNLPHVHRVGENTDHVATATLQLAITELLEDIGLEDEDTIAVTLVPKKGGEGISIEGVEISLADC*

**>Smel_008g312430.1.01_1 PPO3**

MASSFLPLCIHPSFSNTSESSFLPKPSQLFLQRRHNQRFKVSCNANKHEKDNLDVVDRRNVLLGLGGLGAANLAPLTANAAPSPPPDFKTCGIATITADGPPVPYTCCPPPMPSNVNTIPYYKLPSMTKVRIRQPAHTVDEEFIAKYNLAISRMKELDEKEPLNPLGFKQQANIHCAYCNGAYKIGEKVLQVHQSWLFFPFHRWYLYFYERMLGKLIDDPTFALPYWNWDLPKGMRLPPMFDREGSPLYDERRNPQVRNGTVMDLGSFGDQVQTTELQLMSNNLTLMYRHMVTNASCPLLFFGGRYVLGSTQGVQGTIEKIPHTPVHIWVGTKKDSILPNGKKSYGEDMGNFYSAALDPVFYCHHSNVDRMWNEWKQIGGKRRDLSQKDWLDSEFFFYDENKNPYLVKVRDCLDTKKMGYDYAPSSTVWRNFKPNKKNTDGKVNTGSLPSATKIFPIFKLDKAISFSINRPASSRTQQEKNEQEELLTFSYIKYDNREYIRFDVFVNVDKNVKADELDKIEYAGSYTSLPHVHKDGDKDHIATATLQLALTELLEDIGLENEETIAVTLVPKKGGEGLSIGCVEIKLEDC*

**>Smel_008g312420.1.01_1 PPO4**

MSSSSSTLPLCNSKSLFFSFCNSPFLPQPSKLFLQRTRSQRFKVSCNANNVGEHDKNLDAVDRRNVLLGLGGLYGAANLAPLAANAAPIPPPELKTCGRAVVNDTTGELVKYSCCPPIPDDIDSVPYYKFPSMTKLRIRPPAHAVDEEYIAKYQLATSQMRELDKDPFGPIGFKQQANIHCAYCNGAYKAGGKELQVHFSWLFFPFHRWYLYFYERILGSLINDPTFALPYWNWDHPKGMRLPPMFDVEGSSLYDAKRNQSHRNGKIIDLGFFGQETETTELQTMTNNLTLMYRQMVTNAPCPLLFFGNPYPLGTDPKPGMGTIENIPHTPVHIWTGDSPRQPNGEDMGNFYSAGLDPVFYCHHANVDRMWNEWKAIGGKRRDLADKDWLNSEFFFYDENRNPFKVKVRDCLDSKKMGFDYAPMPTPWRNFKPVRRTTSGKANTRSIPPASKVFPTCETRQSDFIFHRQTSFVKDSKAEKNEQEGDTNIRQIQYDDSQYVRFDVFLNVDKTVKALELDQPEFAGSYTSLPHVHGDKDRAPVTFKLAITELLEDNNLEDEESIVITLIPKAGGDGISIQNAVIDLVDC*

**>Smel_008g311990.1.01_1 PPO5**

MSSSSSTTTLPLCTNKSLSSFTNSSFLAKPSQLFLHRSRSQSFKVSCNANNVGEHDKNLDAVDRRNVLLGLGGLYGAANLAPLAANAAPIPPPDLKSCSKAHINPDKEVTYSCCPPIPQDIDSVPYYKFPPMTKLRIRPPAHAVDEEYIAKYQLATSRMRELDKDPFDPLGFKQQANIHCAYCNGAYKVGGKELQVHFSWLFFPFHRWYLYFYERILGSLINDPTFALPYWNWDHPKGMRIPPMFDREGSSLYDEKRNQNHRNGKIIDLGFFGTETQTTELQTMTNNLTYMYRQMVTNAPCPLLFFGNPYPLGTDPSPGMGTIENIPHNPVHIWTGDSPRQPNGEDMGNFYSAGLDPVFYCHHANVDRMWNEWKAIGGKRRDLADKDWLNSEFFFYDENRNPFKVKVRDCLDSKKMGFDYAPMPTPWRNFKPIRKTTSGKANIGSIPPASKVFPIAKLDRAISFSINRPASSRTQAEKNEQEEILTFNKVKYDDSQYVRFDVFLNVDKTVNADELDKAEFAGSYTSLPHVHGDNNTHVTSVTFNLAITELLEDIGLEDEDTIALTLVPKQGGEGISIDNAEIVLVDC*

**>Smel_008g312010.1.01_1 PPO6**

MSSSSATLPLCTNKSLSSFTNSSFLAKPSQLFLHRSRSQSFKVSCNANNVGEHDKNLDAIDRRNVLLGLGGLYGAANLAPLAASAAPIPSPDPKSCSKAHIKPNKEVPYSCCPPPPQDIDSVPYYKFPPMTKLRIRPPAHAVDEEYIAKYQLATSRMRELDKDPFDPLGFKQQANIHCSYCCGAYKVGGKVLQVHSSWLFFPFHRWFLYFYERILGSLINDPTFALPYWNWDHPKGMRIPPMFDHEGSSLYDEKRNQNHRNGKIINLGFSCKETQTTELQTMTNNLTLMYRQMVTNAPCPLLFFGNPYPLGTDPKPGMGTIENIPHNAVHNWTGDQPRQPNGEHMGTFYSAGLDPVFYSHHANVDRMWNEWKAIGGKRRDLADKDWLNSEFFFXFYSAGLDPVFYCHHANVDRMWNEWKAIGGKRRDLADKDWLNSEFFFYDENRNPFKVKVRDCLDSKKMGFDYAPMPTPWRNFKPIRKTTSGKANIGSIPPASKVFPIAKLDRAISFSINRSASSRTQAEKNEQEEILTFNKVQYDDSQCVRFDVFLNVDKTVNADELDKPEFAGSYTSLPHVHGDNNTHVTSVTFKLVITELLEDIGLEDEDTIAVTLVPKEGGEGISIENAEIVLMDC*

**>Smel_008g312490.1.01_1 PPO7**

MASVCNSSTTTLKSPLTSSPSLRSTPKPSQLFLHGKRNQTFKVSCKVTNSNGDQNQNVVETNSVDRRNVLLGLGGMYGVANAIPLAASATPVPPPDLTSCRKAKITETEEVAYLCCAPKPDDMKKVPYYKFPSATKLRLRQPAHAADEEFIAKYNLAISKMKDLDKTEPTNPIGFKQQANIHCAYCNGAYAIDDKVLQVHNSWLFFPFHRWYLYFYERILGKLVDDPTFALPYWNWDHPKGMRLPSMFDRQGTDLYNERRNPQVRNGTVLDLGSFGDQIQTTQLQLMSNNLTLMYRQLVTNAPCPLMFFGAPYVLGNNVEAPGTVEVIPHGPVHVWTGTRAGSILPDGSRSHGEDMGNFYSAGLDPVFYCHHSNVDRMWNEWKAIGGKRRDISHKDWLNSEFFFYDENGNPFRVKVRDCLDTKKMGYDYAPMPTPWRNFKPKTKASSGKVNTSSLPPASKVFPLAKLDKAISFSINRPASSRTQQEKNEQEEMLTFSEIKYDNREYIRFDVFLNADKNLNADELDKAEFAGSYTSLPHVHRAGDTNHIATATLGLAVTELLEDIGLEDENTIAVTLVPKKGGEGISIGGVQITLADC*

**>Smel_008g312460.1.01_1 PPO8**

MTSFSSKILTTPSPFFTKPCQRSQSFNVSCEKKFEANFDRRNVLLGLGGMYGASNLEPLAACASPIPPPDLKSCNRATISEGPEVPYSCCPRKPEDLDNIPYYKLPSMSKLRKRPAAQDVNEEYTAKYQLATKKMKELDDDDPLGFKQQANIHCAYCNNAYKIDGKVLQVHQSWLFFPFHRWYLYFYERILGSLINDPTFALPYWNWDHPKGMCLPPMFDVEGSSLYDERRNPHVRNGAIIDLGSFGDEVKTAELQMRTNNLTLMYRQMITNAPCPSQFFGARYVLGTDPKGQGTIENIPHTPVHIWTGTVRGPNNLGNGATSYGEDMGNFYSAGLDPVFFCHHANVDRMWNIWKQLGGKRRDLMDNDWLNSEFFFYDENRNPYRVRVRDCLDSKKMGYVYAPMPTPWRNFKPTKKSQVGKVSSNSINPASKVFPLAKLDRAISFSINRQRDKNEQEEVLTFKGIKYDNSKYIRFDVFVNAEESVNADELDKIEFVGSYVSMAPLVHGHKMESDESFQVAIREVLEDNGLADDDTITVTLVPKKGGQLLSIQSVDIEFVTG*

**>Smel_008g312520.1.01_1 PPO9**

MASNFLITSCPIISSSPSKFSQSPSKIFVSAKCNGNFKVNCSNNDNEGKSFSEKLDRRNVLVGLGGLYGASNFIGVANEPFALGAPVPPPDLSTCNTASLPDGSPVPFTCCPPLPKDLSNIPTYKLPDVSSKLKIRPAAHNVTQEYITKYSIAIQKMKSLDKDDPLNFMQQANIHCAYCNSGYKELGFPGVPLQVHFSWLFFPFHRWYLYFFERILGSLIGDDTFALPFWNYDSKVGMQLPSLYNDVNSPLYDPNRNQNHLPPNVVDIGFTTIDLDVSDQQKINNNLTMMYRQMITNAPCPQLFFGNPIRGGEQPIRGMGTIENIPHNAVHRWVGNPNNKFRENMGTFYSAARDPIFYAHHANIDRMWTIWKTLGAKRRDFTDRDWLDSAFLFYDETRTLVKVTVQDCINNEKLGYKYENVPIPWKNYRPVPRKQKVKKNANKVKPSTEIFPSTLKKTLSFSIKRPNISRTQQDKDTQEELLVFNNMTFDENEYIRFDVFINEDEGVKAKVLDRIEYVGSFANLPHVHNGGQNTSSATPAIMSLAITEILEDLGLEDEEEIVVVVVPHSGGKEITIGSVEINTLACAN*

**>Smel_000g064350.1.PPO10**

MSSVPLHTTNTISSSTTTTFSNLHPSPFFGKTPKISSIKKHNGHRNFKVSCKSTDENNNHEHNSPIDISKKSTNSSSNIIDRRNVLLGLGGLYGASTLVGGLPFAFAAPVNGPDVNKCGAADLPPGAAPVNCCPPMTGNIIDFQLPSSTTIRTRPAAHSVDSAYIEKFNRGIQLMKELPDNDPRSFKQQANVHCAYCDGAYDQLGFPNTELQVHSSWIFLPFHRCYLYFFERILGSLINDPTFAMPFWNWDHPDGMRLPGMYANSTSALYDRLRDRRHQPPIMVDLDFNGTDPNISDSQQTSQNLTNMYRQMISLGRTPETFLGDPYRAGGLPGGGGSLENMAHGSVHVWTGDRTQPNFENMGDFYSAARDPIFYAHHSNIDRLWSVWKTLGGRRQDFTDPDFLNTSFLFYDEKAQMVRIRVRDVLDSSKMGYVYQNVTNPWINSRPTPRVSRALSSIRRLVEARAADDNVMTFPRPKDVFPTKLDHVIKVMVKRPNKNKRNKKEKNEKEEILVVEGLEVETDVFVKFDVLINDEDETLTSPGNAEFAGSFVNVPHHRHGKGDKNSKRKTKLKLAITELLEDLDAENDENVLVTFVPKNGSGAVKIGNVKIVLED*
