# Supplementary figures and images for: Simultaneous CRISPR/Cas9 Editing of Three PPO Genes Reduces Fruit Flesh Browning in Solanum melongena L
Source: Front Plant Sci. 2020 Dec 3;11:607161. doi: 10.3389/fpls.2020.607161 (PMC7744776; doi:10.3389/fpls.2020.607161)

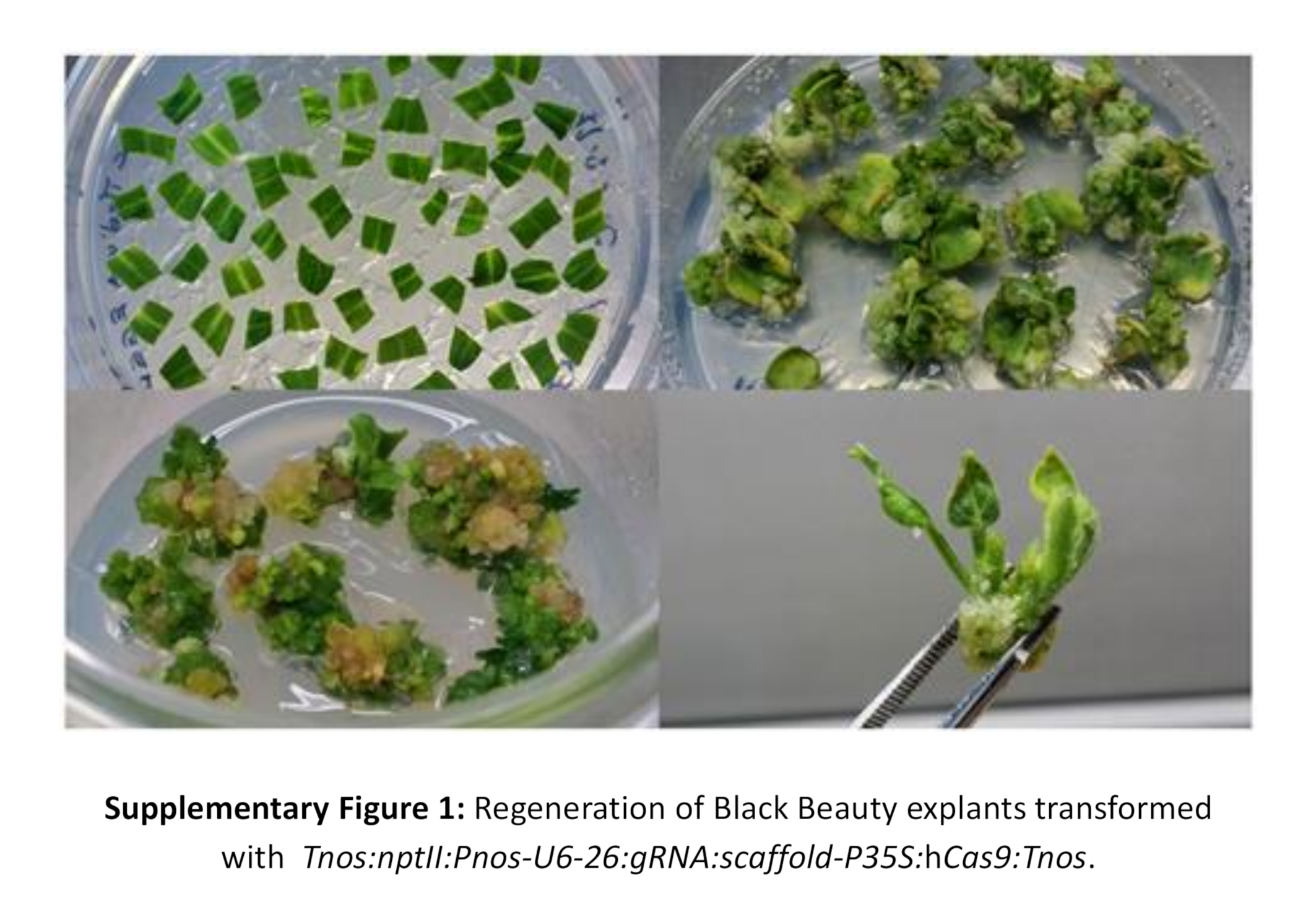

Supplement: Supplementary file 8 [file Image_1.TIFF]

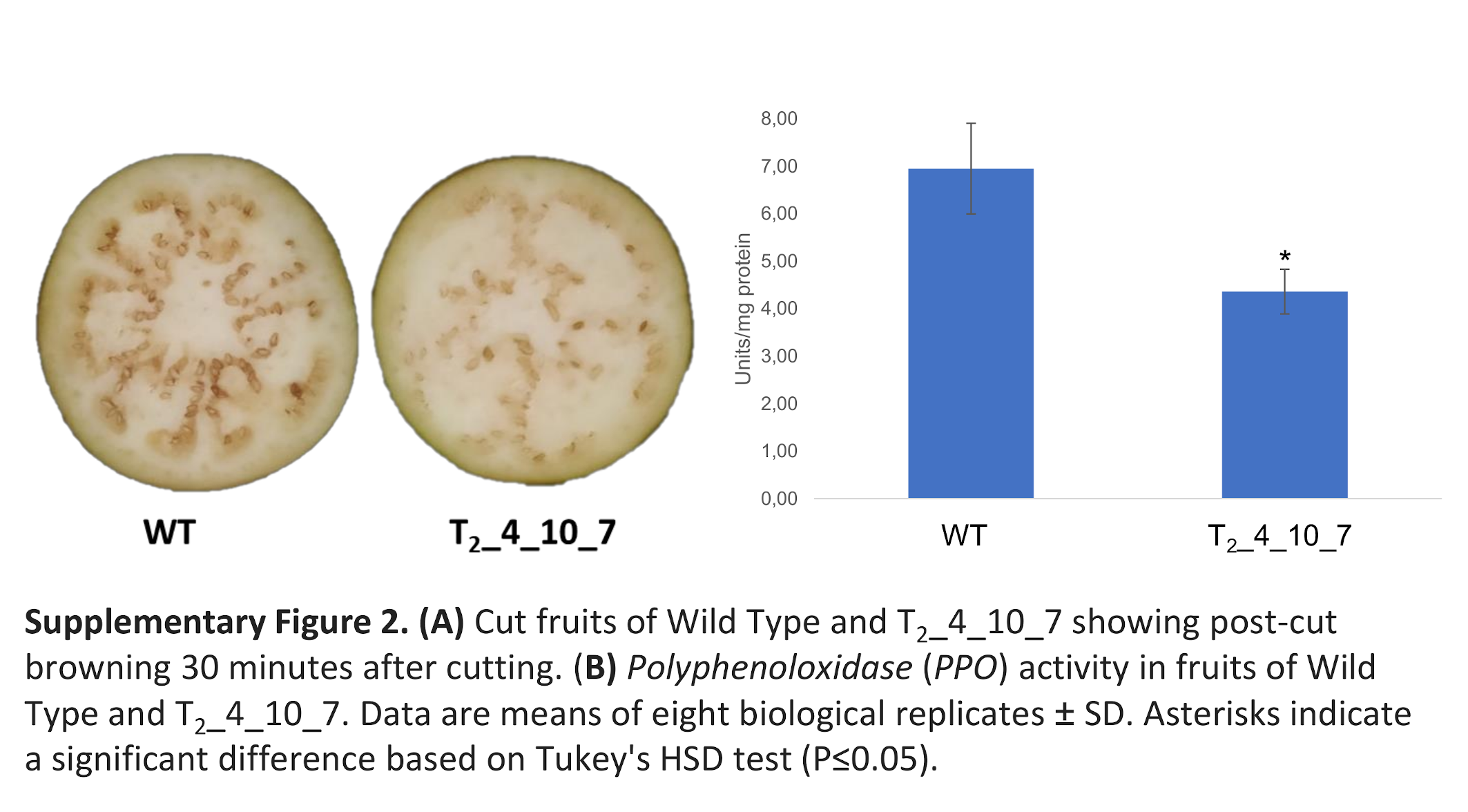

Supplement: Supplementary file 9 [file Image_2.TIFF]
